# Disautonomia: Uma Condição Esquecida – Parte 1

**DOI:** 10.36660/abc.20200420

**Published:** 2021-04-08

**Authors:** Eduardo Arrais Rocha, Niraj Mehta, Maria Zildany Pinheiro Távora-Mehta, Camila Ferreira Roncari, Alan Alves de Lima Cidrão, Jorge Elias

**Affiliations:** 1 Universidade Federal do Ceará Hospital Universitário Walter Cantídio Faculdade de Medicina da UFC FortalezaCE Brasil Hospital Universitário Walter Cantídio da Universidade Federal do Ceará (UFC) - Programa de Pós-graduação em Ciências Cardiovasculares da Faculdade de Medicina da UFC, Fortaleza, CE - Brasil; 2 Universidade Federal do Paraná CuritibaPR Brasil Universidade Federal do Paraná, Curitiba, PR - Brasil; 3 Clínica de Eletrofisiologia do Paraná CuritibaPR Brasil Clínica de Eletrofisiologia do Paraná, Curitiba, PR - Brasil; 4 Universidade Federal do Ceará Faculdade de Medicina Departamento de Fisiologia e Farmacologia FortalezaCE Brasil Departamento de Fisiologia e Farmacologia - Faculdade de Medicina da Universidade Federal do Ceará (UFC), Fortaleza, CE - Brasil; 5 Faculdade de Medicina da UFC FortalezaCE Brasil Programa de Pós-graduação em Ciências Cardiovasculares da Faculdade de Medicina da UFC, Fortaleza, CE - Brasil; 6 Serviço de Eletrofisiologia do Vitória Apart Hospital VitóriaES Brasil Serviço de Eletrofisiologia do Vitória Apart Hospital, Vitória, ES - Brasil

**Keywords:** Disautonomia, Síncope, Hipotensão Ortostática, Síndrome da Fadiga Crônica, Amiloidose, Doença de Chagas, COVID-19, Neuropatia Autonômica Cardiovascular, Hipersensibilidade do Seio Carotídeo, Diabetes Mellitus

## Abstract

O termo disautonomia abrange um conjunto de condições clínicas com características e prognósticos distintos. Classificam-se em síndromes reflexas, síndrome postural ortostática taquicardizante (SPOT), síndrome da fadiga crônica, Hipotensão Ortostática Neurogênica (HON) e a Síndrome da hipersensibilidade do seio carotídeo. As síndromes reflexas (vasovagal) não serão discutidas neste artigo.

1. As síndromes reflexas (vasovagal) são, na maioria das vezes, benignas, e ocorrem usualmente em pacientes sem doença intrínseca do sistema nervoso autônomo (SNA) ou do coração. Por isso, geralmente são estudadas separadamente.
2. O termo neuropatia autonômica cardiovascular (NAC) é o mais utilizado na atualidade para definir as disautonomias com comprometimento do sistema nervoso autônomo cardiovascular simpático e/ou parassimpático. Pode ser idiopática, como a atrofia multissistêmica ou a falência autonômica pura, ou secundária a patologias sistêmicas como diabetes mellitus, doenças neurodegenerativas, doença de Parkinson, síndromes demenciais, insuficiência renal crônica, amiloidose, podendo também acometer idosos.
3. A presença de neuropatia autonômica cardiovascular (NAC) implica em maior gravidade e pior prognóstico em diversas situações clínicas.
4. A detecção de hipotensão ortostática (HO) é um sinal tardio e significa maior gravidade no contexto das disautonomias, definida como hipotensão ortostática neurogênica (HON). Deve ser diferenciada das hipotensões por hipovolemia ou medicamentosas, chamadas de hipotensão ortostática não neurogênica (HONN).
5. A HO pode decorrer de causas benignas, como a hipovolemia aguda, crônica, ou ao uso de diversos fármacos. Esses fármacos podem, entretanto, apenas desmascarar quadros subclínicos de disautonomia. Deve-se reavaliar todos os fármacos de pacientes com quadros disautonômicos.
6. O diagnóstico preciso de NAC e a investigação do envolvimento de outros órgãos ou sistemas é de extrema importância na suspeita clínica de uma pandisautonomia.
7. No diabético, além da idade e do tempo de doença, outros fatores estão associados a maior ocorrência de NAC, como descontrole glicêmico, hipertensão, dislipidemia e obesidade. Entre os pacientes diabéticos, 38–44% podem evoluir com disautonomia, com implicações prognósticas e maior mortalidade cardiovascular. Nas etapas iniciais da DM, a disfunção autonômica envolve o sistema parassimpático, posteriormente o simpático e mais tardiamente manifesta-se com hipotensão ortostática.
8. Os testes de Valsalva, respiratório e ortostático (30:15) são os métodos de padrão ouro para o diagnóstico de NAC. Eles podem ser associados aos testes de variabilidade RR no domínio do tempo, e principalmente da frequência, para aumento da sensibilidade (protocolo dos 7 testes). Esses testes podem detectar alterações iniciais ou subclínicas e avaliar a gravidade e o prognóstico.
9. O teste de inclinação (*tilt test*) não deve ser o exame de escolha para investigação de NAC em fase inicial, pois detecta casos em fases mais avançadas. A resposta no tilt com padrão disautonômico (queda gradativa da pressão arterial sem aumento da frequência cardíaca) pode sugerir NAC.
10. O tratamento dos pacientes em fases moderadas a avançadas das disautonomias é bastante complexo e muitas vezes refratário, necessitando de avaliação especializada e multidisciplinar. Não há cura para a maioria das disautonomias em fase tardia.
11. Os pacientes com HON podem evoluir com hipertensão supina em mais de 50% dos casos, representando um grande desafio terapêutico. O risco imediato e as consequências da HO devem ter preferência sobre os riscos mais tardios da hipertensão supina e valores maiores que 160/90 mmHg são toleráveis. Medidas como dormir com a cabeceira elevada (20–30 cm), não levantar à noite, uso de anti-hipertensivo de ação curta noturna para casos mais severos, como a losartana, captopril, clonidina ou adesivos de nitratos, podem ser necessários e efetivos em alguns casos.
12. As medidas preventivas como cuidados posturais, boa hidratação, maior ingesta de sal, uso de meias e cintas abdominais compressoras, refeições fracionadas, atividade física supervisionada principalmente sentada, deitada ou exercícios na água são etapas importantes no tratamento.
13. Diversos fármacos podem ser usados para HON sintomática, principalmente a fludrocortisona, a midodrina e a droxidopa. Esses últimas não estão disponíveis no Brasil. O risco de exacerbação ou desencadeamento de hipertensão supina deve ser considerado.
14. A síndrome da fadiga crônica representa uma forma de disautonomia e tem sido renomeada como doença sistêmica de intolerância ao exercício, com novos critérios diagnósticos: 1 - Fadiga inexplicada, levando a incapacidade para o trabalho por mais que 6 meses; 2 - Mal-estar após exercício; 3 - Sono não reparador; 4 - Mais um dos seguintes achados: comprometimento cognitivo ou intolerância ortostática. Várias patologias na atualidade têm evoluído com fadiga crônica, sendo denominadas de doenças crônicas associadas a fadiga crônica.
15. A síndrome postural ortostática taquicardizante (SPOT), outra forma de apresentação das síndromes disautonômicas, é caracterizada por elevação sustentada da frequência cardíaca (FC) ≥30 bpm (≥40 bpm se <20 anos) ou FC ≥120 bpm, nos primeiros 10 minutos em posição ortostática ou durante o *tilt test*, sem hipotensão ortostática clássica associada. Pode ocorrer leve redução na pressão arterial. Os sintomas manifestam-se ou pioram em posição ortostática, sendo comuns a tontura, fraqueza, pré-síncope, palpitações, além de outros sintomas sistêmicos.

**Síndromes Vasovagais x Disautonomia**

As síndromes vasovagais são situações clínicas distintas das neuropatias autonômicas cardiovasculares, pois não representam doenças intrínsecas no sistema nervoso autônomo (SNA), sendo decorrentes de mecanismos reflexos, transitórios, benignos, tendo, portanto, prognóstico favorável.

## Disautonomia: uma condição frequente e subdiagnosticada

O sistema nervoso autônomo (SNA) regula importantes funções nos diversos sistemas orgânicos como cardiovascular, digestório, gênito-urinário e sudomotor. Suas disfunções podem determinar diversas manifestações clínicas, algumas debilitantes e graves. Diversas patologias podem comprometer o SNA e determinar sintomatologia, aumentando os riscos de síncope, quedas e de maior mortalidade cardiovascular. Em virtude das diferentes manifestações clínicas e da pouca familiaridade dos profissionais, a disautonomia costuma ser frequentemente subdiagnosticada, sendo reconhecidas em etapas mais avançadas, com sintomas já debilitantes, incapacitantes e com pior prognóstico.

O termo neuropatia autonômica cardiovascular (NAC) significa envolvimento do sistema nervoso autônomo, relacionado às funções cardiovasculares. A diabetes mellitus (DM) representa a forma mais comum e estudada das NAC e serve de modelo de compreensão e investigação para diversas outras patologias.^1^^,^^2^

Na população diabética, é conhecida como neuropatia autonômica cardiovascular diabética, tendo prevalência de 20% em pacientes com DM, sendo de até 54% no tipo I (DM1) e 46% no tipo II (DM2), na faixa entre os 40 e 70 anos de idade. No diabético, além da idade e tempo de doença, outros fatores estão associados a maior risco de NAC, como descontrole glicêmico, hipertensão, dislipidemia e obesidade. Nas etapas iniciais da DM, a disfunção autonômica envolve o sistema parassimpático, posteriormente o simpático e mais tardiamente, evoluem com hipotensão ortostática.

O sistema nervoso autônomo cardiovascular modula a frequência cardíaca, os volumes diastólico e sistólico, o intervalo QT e a resistência vascular sistêmica. Seu comprometimento está relacionado à maior morbimortalidade cardiovascular.

O objetivo desta revisão é oferecer informações relevantes das diferentes formas de disfunções autonômicas, suas manifestações clínicas, metodologias diagnósticas, terapêuticas e implicações prognósticas. Enfatizamos a importância do diagnóstico, de sua distinção das síndromes reflexas vasovagais e a necessidade de maior difusão das informações dessas patologias, já que é pouco lembrada na prática clínica geral. As síndromes reflexas vasovagais não serão abordadas neste capítulo.

Foram consideradas para elaboração desta revisão, diversas diretrizes, como: Diretrizes de Neuropatia Autonômica Cardiovascular (NAC), Consenso de Hipotensão Ortostática Neurogênica e Hipertensão Supina, Diretrizes de Síncopes, Diretrizes de NAC no Diabético, Diretrizes de Testes Cardiovasculares em Neuropatia Autonômica, o Consenso de Investigação de Disfunção Autonômica em Estudos de Pesquisa Humana, Consenso no Diagnóstico e Tratamento da Síndrome Postural Ortostática Taquicardizante e Taquicardia Sinusal Inapropriada, dentre outros estudos. Discussões entre especialistas da Sociedade Brasileira de Arritmias Cardíacas foram incluídas, considerando a carência de grandes estudos em diversos tópicos abordados neste trabalho.^1^^–^^20^

## Fisiologia do Sistema Nervoso Autônomo

O sistema nervoso autônomo (SNA) possui um papel importante no controle das funções viscerais através das subdivisões simpática e parassimpática.

O SNA propicia ajustes neurovegetativos para a expressão de comportamentos motivados ou respostas compensatórias frente a estímulos internos e externos para, juntamente com o sistema endócrino, promover a manutenção da homeostase. O termo “sistema nervoso autônomo” foi proposto por Langley em 1898, uma vez que as nomenclaturas utilizadas até então tinham diferentes conotações e eram imprecisas quanto às funções recentemente descobertas desse sistema.^20^

Para facilitar a compreensão, o SNA é comumente analisado quanto aos seus aspectos anatômicos, neuroquímicos e funcionais. A organização básica envolve dois grupos neuronais, arranjados em série e conectados por uma sinapse química. O segundo neurônio dessa série está completamente fora do sistema nervoso central e o seu corpo celular está localizado nos gânglios autonômicos, de onde partem projeções axonais, que vão inervar os órgãos-alvo, sendo denominados neurônios pós-ganglionares.^21^

Já os neurônios, que enviam as projeções axonais do sistema nervoso central para os gânglios, fazendo sinapse com os corpos celulares presentes nessas estruturas, são denominados neurônios pré-ganglionares.

A diferença anatômica entre SNA simpático e parassimpático diz respeito à localização dos corpos celulares dos neurônios pré-ganglionares, sendo que os neurônios pré-ganglionares simpáticos estão localizados nos segmentos torácicos e lombares da medula espinhal, e os parassimpáticos, no tronco encefálico e nos segmentos sacrais da medula espinhal.

Em relação à neuroquímica, todos os neurônios pré-ganglionares são colinérgicos e usam acetilcolina como neurotransmissor. Embora haja algumas exceções, os neurônios pós-ganglionares parassimpáticos liberam acetilcolina no órgão-alvo, enquanto os simpáticos liberam noradrenalina.

As células da medula adrenal são homólogas aos neurônios pós-ganglionares simpáticos e secretam principalmente adrenalina e, em menor proporção, noradrenalina diretamente na corrente sanguínea, em resposta à estimulação por neurônios pré-ganglionares simpáticos.

Finalmente, o sistema nervoso simpático e o parassimpático divergem quanto às respostas desencadeadas nos órgãos-alvo. Algumas poucas estruturas recebem inervação única, enquanto a maioria dos órgãos recebem inervação dupla. As respostas induzidas pela estimulação do SNA simpático e parassimpático podem ser antagônicas ou cooperativas.

**Figura 1:**
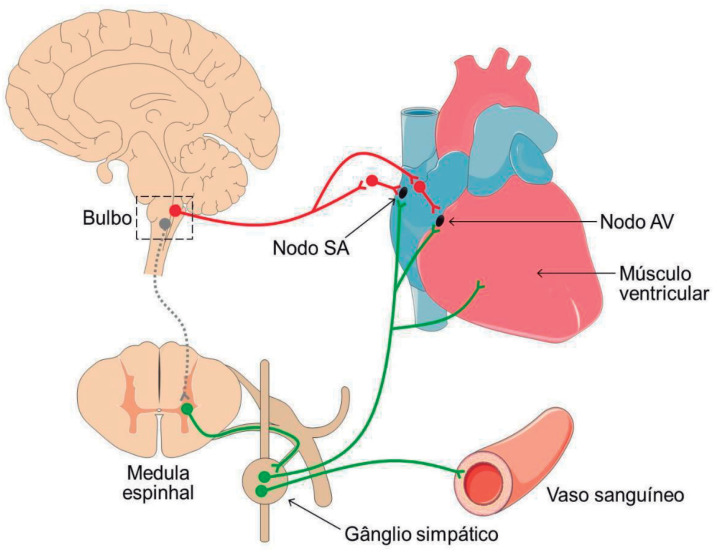
Inervação do coração e dos vasos sanguíneos pelo SNA simpático e parassimpático. Os neurônios parassimpáticos estão representados em vermelho e os neurônios simpáticos, em verde. Nodo SA — nodo sinoatrial; nodo AV — nodo atrioventricular. Para facilitar a visualização, foi representado um único segmento medular esquemático e as imagens não estão representadas na mesma escala gráfica.

Como mostrado na figura 1, os vasos sanguíneos sistêmicos são inervados pelo SNA simpático. A maior ativação dos receptores α^1^-adrenérgicos pelo aumento do tônus simpático ou liberação de adrenalina pela glândula adrenal causa vasoconstrição na maioria dos vasos sanguíneos sistêmicos, especialmente nos vasos das vísceras abdominais, um importante leito de resistência com grande influência sobre a determinação da pressão arterial (PA).

Em contrapartida, a redução do tônus simpático ou dos níveis plasmáticos de adrenalina resultam em vasodilatação. Os vasos sanguíneos coronários particularmente expressam receptores β^2^ e sofrem vasodilatação em resposta à adrenalina.

O coração é inervado pelos sistemas simpático e parassimpático (Figura 1). A inervação parassimpática cardíaca é direcionada para os nodos sinoatrial (SA) e atrioventricular (AV) e a acetilcolina se liga aos receptores colinérgicos muscarínicos M^2^, expressos nas células dos nodos, induzindo efeito cronotrópico negativo. Por outro lado, o SNA simpático inerva tanto os nodos SA e AV quanto o músculo ventricular. A noradrenalina induz efeitos cronotrópico e inotrópico positivos por ação em receptores β^1^-adrenérgicos.^22^

Todas as células cardíacas, em princípio, apresentam a propriedade elétrica de automatismo; no entanto, em condições fisiológicas, as células do nodo SA apresentam despolarização espontânea em maior frequência e assumem o controle dos batimentos cardíacos, sendo consideradas o marca-passo cardíaco.

Após o bloqueio farmacológico dos receptores muscarínicos e β-adrenérgicos, a frequência cardíaca intrínseca gerada pelo nodo sinoatrial é de aproximadamente 100 batimentos por minuto, sugerindo que existe um predomínio da influência parassimpática sobre o coração.^23^ Para ajustes da PA, o tônus simpático e parassimpático para o coração e vasos sanguíneos são frequentemente modificados pelo barorreflexo.

A pressão arterial (PA) é constantemente monitorada pelos barorreceptores de alta pressão (receptores de estiramento) presentes no arco aórtico e no seio carotídeo, que enviam sinalização através do nervo vago e glossofaríngeo, respectivamente, para o núcleo do trato solitário (NTS), localizado na porção dorsomedial do bulbo.^24^

Em situações de elevação da PA, os barorreceptores são mais ativados e, por mecanismos barorreflexos, ocorre um aumento do tônus parassimpático e redução do tônus simpático para coração e vasos sanguíneos. O aumento dos disparos dos barorreceptores ativa o NTS que, por sua vez, ativa o núcleo ambíguo (NA), núcleo bulbar onde se encontram os corpos celulares dos neurônios pré-ganglionares parassimpáticos, resultando em aumento do tônus parassimpático. Paralelamente, o NTS também ativa o bulbo caudal ventrolateral (BCVL), região que emite projeções inibitórias para o bulbo rostral ventrolateral (BRVL). Os neurônios do BRVL são considerados pré-simpáticos, porque se projetam para a coluna intermediolateral da medula espinhal e fazem sinapse com os corpos celulares dos neurônios pré-ganglionares simpáticos. Assim, a maior atividade da BCVL resulta em inibição da BRVL e consequente redução do tônus simpático.

Por outro lado, a menor atividade dos barorreceptores durante queda da PA resulta em: 1) menor ativação do NA e, portanto, redução do tônus parassimpático; e 2) menor ativação do BCVL e, consequentemente, maior atividade do BRVL e aumento do tônus simpático para o coração e vasos sanguíneos.

Alterações no funcionamento normal do mecanismo barorreflexo podem desencadear condições patológicas denominadas disautonomias, como a hipotensão ortostática neurogênica, por exemplo. A mudança da posição supina para a ortostática aumenta a resistência gravitacional ao retorno venoso, resultando em redução do volume diastólico final e, consequentemente, do volume sistólico (VS), observada em diversas patologias.

A PA é diretamente proporcional à resistência periférica total e ao débito cardíaco, sendo esse último o volume de sangue bombeado pelo coração por minuto, ou seja, VS multiplicado pela frequência cardíaca (FC).

Assim, a redução do VS após mudança para a posição ortostática induz hipotensão. Em indivíduos saudáveis, essa hipotensão é transitória porque os mecanismos barorreflexos são rapidamente ativados e causam aumento de força de contração e FC e vasoconstrição sistêmica, respostas compensatórias que normalizam a PA. Já em indivíduos que apresentam disautonomia, pode ocorrer hipotensão prolongada, denominada hipotensão ortostática neurogênica (HON).

**Atrofia Multissistêmica (AMS) - Síndrome de Shy-Dragger**

A síndrome completa consiste em hipotensão ortostática, incontinência urinária e fecal, perda de sudorese, atrofia da íris, paralisia ocular externa, rigidez, tremores, perda de movimentos, impotência, achados de bexiga atônica e perda de tônus retal, fasciculações, atrofia de músculos distais e evidência de lesões neuropáticas. A data de início é entre a 5 e a 7ᵃ década de vida.

## Fisiopatologia e as apresentações clínicas

Diversos mecanismos fisiopatológicos têm sido descritos nas alterações do sistema nervoso autônomo (SNA). Eles podem variar dependendo das etiologias específicas, como no diabetes ou na amiloidose. Várias situações, entretanto, têm seus mecanismos causais desconhecidos.

Apesar de outros neurotransmissores serem importantes na regulação das respostas cardiovasculares, a liberação de noradrenalina nas terminações nervosas pós ganglionares simpáticas é o mais importante mediador da rápida regulação cardiovascular necessária no equilíbrio pressórico e perfusão cerebral. A hipotensão ortostática neurogênica representa uma deficiência na responsividade deste neurotransmissor à mudança postural.

Diferente das síndromes reflexas ou vasovagais, nos quadros de disautonomia, os reflexos de aumento na frequência cardíaca precedendo o quadro clínico e a bradicardia concomitante com a hipotensão não são observados.

Na diabetes mellitus, ocorrem alterações metabólicas e vasculares que podem justificar o dano neurológico. A hiperglicemia, o acúmulo de sorbitol, de frutose e de produtos finais da glicação avançada, com ligações a receptores nas células endoteliais e musculares lisas do *vaso-nervorum* das células de Schwann e dos macrófagos, podem contribuir para o dano neurológico. O estresse oxidativo levando à depleção das enzimas antioxidantes celulares e ativação da cascata de inflamação, com deterioração de organelas celulares, principalmente a nível mitocondrial, são outros mecanismos que culminam em oclusão vascular, disfunção endotelial e neuroinflamação, determinando toxicidade e morte neuronal.^25^^–^^30^

Nas sinucleinopatias, entidade que engloba a doença de Parkinson, a demência de corpos de Lewy, a falência autonômica pura (síndrome de Bradbury-Eggleston) e a atrofia multissistêmica (síndrome de Shy-Dragger) ocorrem o depósito intracelular e a agregação de uma proteína chamada alfa-sinucleína em diversas regiões do sistema nervoso central e periférico.^19^^,^^31^^,^^32^

A atrofia multissistêmica (AMS)^32^ uma forma idiopática mais grave e rara, descrita em 1960, apresenta-se sob duas formas: 1. Parkinsonismo, na qual observa-se rigidez muscular e bradicinesia (diferente da clássica doença de Parkinson, na qual predominam tremores; 2. Cerebelar, cuja manifestação é a ataxia. Ambas as formas têm envolvimento do sistema nervoso autônomo.^8^ Imagens na ressonância magnética nuclear cerebral demonstram atrofia cerebelar, da ponte ou dos pedúnculos cerebrais, ou hipersinal na ponte, conhecido como sinal da cruz, que pode ocorrer mais tardiamente. As dosagens de catecolaminas são usualmente normais, por ser uma polineuropatia autonômica pré-ganglionar.

Na falência autonômica pura, de etiologia idiopática, descrita em 1925 e conhecida como uma polineuropatia autonômica pós-ganglionar, os sintomas são graduais, progressivos, podendo chegar a quadros severos e debilitantes, com importante comprometimento cardiovascular, hipotensão ortostática grave, com envolvimento dos sistemas gênito-urinário, digestório e sudomotor.

Por não apresentarem sintomas neurodegenerativos centrais, os exames de imagem cerebral na falência autonômica pura são normais e as dosagens de catecolaminas plasmáticas são normais ou baixas, porém não apresentam incremento adequado (>50%) com a ortostase, devido à denervação simpática periférica difusa.

Algumas toxinas podem ser fatores causais, como intoxicações por chumbo, tálio, arsênio, ou uso de alguns fármacos como quimioterápicos da classe da cisplatina ou dos alcaloides da vinca, antiarrítmicos como amiodarona, ou deficiências vitamínicas como a da vitamina B12.

Casos raros de origem familiar podem ocorrer, como a neuropatia sensorial e autonômica hereditária (HSAN). São divididas em: HSAN tipo I, de forma mais leve, iniciando-se na vida adulta, com envolvimento sensorial e autonômico distal, com ulcerações nos pés; HSAN tipo II, mais rara, com início na infância, com comprometimento mais difuso e severo.^8^^,^^19^^,^^31^^,^^33^

As etiologias autoimunes podem justificar diversas apresentações clínicas agudas e subagudas de pandisautonomias, com algumas similaridades com a síndrome de Guillain-Barré (SGB). Entretanto, nas pandisautonomias agudas, as fibras somáticas são geralmente poupadas, diferente da SGB. Algum grau de disfunção autonômica está presente também na maioria dos casos da SGB.^31^^,^^34^^,^^35^

### Amiloidose

A amiloidose pode se apresentar nas seguintes formas:

1. Na forma mais comum, conhecida como de cadeias leves (AL) ou amiloidose primária, observa-se uma proliferação anormal clonal de células plasmáticas. Inicialmente, ocorre uma neuropatia periférica sensitiva distal, progredindo para fibras largas, com posterior falência autonômica de múltiplos órgãos acometidos, como sistema digestório, incluindo esôfago e intestino, sudomotor, com anidrose alternada com sudorese compensatória, envolvimento renal e síndrome nefrótica e comprometimento cardíaco, com insuficiência cardíaca, arritmias e morte súbita. Na avaliação autonômica, pode-se constatar comprometimento dos sistemas simpático e parassimpático.
2. Na forma da amiloidose familiar (AF), também chamada de paramiloidose ou doença de Corino Andrade,^36^^,^^37^ popularmente conhecida como doença dos pezinhos, apresenta-se na forma autossômica dominante, descrita originalmente pelo professor Português Dr. Corino de Andrade, em 1952. Tem maior incidência entre os 20 e 40 anos, evoluindo para óbito com 10–12 anos de evolução.
  Tem fenótipo variável, dependendo da região geográfica e da mutação. Foram descritas diversas formas como: a portuguesa (tipo I) ou de Andrade, a Rukovina ou tipo Indiana (tipo II), a de van Alien (tipo III) e o tipo Finlandês (tipo IV). No Brasil, foram descritas algumas formas dessa patologia.^38^
  A mutação no gene da transtirretina (TTR) é a mais conhecida e estudada, tendo diversas mutações descritas neste gene. Tem seu início com sintomas de neuropatia periférica, podendo evoluir para graves manifestações de disfunção autonômica generalizada, além de sintomas cardiológicos, neurológicos (polineuropatia periférica sensitivo-motora), visuais, gênito-urinários, renais e gastrointestinais. A detecção precoce é extremamente importante, visando o tratamento e evitando a progressão. O transplante hepático antes da doença apresentar-se avançada pode modificar a evolução. Novas drogas promissoras têm sido lançadas como o Tafamidis (estabilizadoras da TTR), já disponível no Brasil, e o Inotersen.
3. A forma secundária (forma AA) decorre de patologias crônicas como artrite reumatoide, osteomielite, tuberculose, insuficiência renal, e seu quadro evolutivo dependerá do controle da doença de base.

A amiloidose cardíaca é causada principalmente pela AL ou AF tipo transtirretina (ATTR) ou por deposição de proteínas transtirretina tipo selvagem (*wild-type transthyretin protein),* previamente chamada de amiloidose cardíaca senil. Depósitos de TTR foram observados em 16% dos pacientes com estenose aórtica degenerativa e em até 17% dos pacientes com insuficiência cardíaca com fração de ejeção preservada. O prognóstico após acometimento cardíaco é ruim, com a sobrevida variando entre 2,5 e 3,6 anos. O ecocardiograma, com aumento importante da espessura da parede do ventrículo esquerdo (>14 mm), apesar da baixa voltagem no eletrocardiograma, pode sugerir o diagnóstico, sendo complementado pela ressonância magnética nuclear cardíaca e a cintilografia com pirofosfato de tecnésio.^39^

No estudo randomizado ATTR-ACT de avaliação da segurança e eficácia do Tafamidis em pacientes com amiloidose cardíaca, foram observadas redução de todas as causas de mortalidade e internações hospitalares após 30 meses de seguimento, passando a ser indicado nessa patologia para insuficiência cardíaca classe funcional (CF) I, II e III NYHA (*New York Heart Association*), principalmente nas fases precoces. Essa foi a primeira terapia a mostrar melhora na sobrevida desses pacientes.^40^

Em muitos casos de disautonomias, relatos de infecções virais recentes são identificadas, principalmente por herpes-vírus, Epstein-Barr e Coxsackie. Autoanticorpos antirreceptores gangliônicos de acetilcolina (AChr) foram encontrados em 50% dos pacientes com FAP, em 7% dos pacientes com SPOT e em 0% nos controles. A ausência desses anticorpos não afasta o diagnóstico. Relatos de casos demonstraram sucesso terapêutico com aplicação de imunoglobulinas em algumas dessas situações clínicas.^31^^,^^34^^,^^35^^,^^41^^–^^43^

Nas síndromes paraneoplásicas, mais comumente nos carcinomas de pequenas células pulmonares, autoanticorpos, principalmente o anti-Hu ou ANNA-1, usualmente estão presentes, e as apresentações clínicas costumam ser agudas ou subagudas.

A teoria autoimune é reforçada pelo aparecimento de sintomas após quadros virais, estados febris, após vacinação e em pacientes com doenças autoimunes prévias, como tireoidite de Hashimoto, doença celíaca e lúpus eritematoso sistêmico.

Estudos demonstraram que a teoria autoimune pode ser o mecanismo fisiopatológico das formas “idiopáticas” de algumas síndromes disautonômicas, como a falência autonômica pura (FAP), SPOT ou a síndrome da fadiga crônica.^43^

Anticorpos antirreceptores colinérgicos nicotínicos também foram descritos. Recentemente, autores demonstraram o mecanismo pelo qual os autoanticorpos causam vasodilatação e taquicardia. Esses achados podem ter implicações terapêuticas importantes. Na presença de anticorpos antirreceptores de acetilcolina, o uso de fármacos como a piridostigmina podem ser benéficos. Já na presença de anticorpos adrenérgicos, os betabloqueadores poderiam ser a melhor escolha.

### Doença de Chagas

A disautonomia cardíaca está bem estabelecida na doença de Chagas (DCh), na qual a denervação anatômica e anormalidades funcionais têm sido descritas em estudos *in vivo, post mortem* e experimentais.^44^^-46^ Os trabalhos originais de Carlos Chagas já chamavam a atenção para a ausência de resposta cronotrópica à atropina nos pacientes chagásicos.^47^ Além da denervação, outras alterações no sistema nervoso autônomo, como ganglionite, neurite, fibrose, atrofia e fragmentação de fibras especializadas também foram relatadas.^48^

O comprometimento parassimpático pode ser detectado em todas as formas da DCh, incluindo a fase indeterminada e independente da função ventricular esquerda.^49^^,^^50^ Esses dados foram corroborados por uma metanálise que incluiu sete estudos que avaliaram a modulação autonômica cardíaca, usando a variabilidade R-R durante a manobra de Valsalva.^51^

Estudos com metaiodobenzilguanidina I-^123^ (^123^I-MIBG) detectaram disfunção simpática em pacientes chagásicos com forma indeterminada e sem disfunção sistólica do ventrículo esquerdo.^52^^,^^53^ A cintilografia com ^123^I-MIBG também foi utilizada para avaliar a presença e magnitude da disfunção simpática em pacientes com cardiopatia chagásica e disfunção ventricular (FE≤45%). Os autores observaram uma baixa na captação de ^123^I-MIBG, indicando uma disfunção dos receptores simpáticos e perda da integridade das fibras simpáticas pré-sinápticas.^52^

Um aspecto que demanda mais elucidação é o papel dos mecanismos imunomediados na cardiopatia chagásica. De fato, vários estudos demonstraram a presença de anticorpos que reagem com os receptores M2 muscarínicos cardíacos e receptores adrenérgicos B1 no soro de pacientes chagásicos assintomáticos.^48^^,^^54^

Esses autoanticorpos poderiam desempenhar um papel na patogênese da miocardite chagásica, explicando a neuromiopatia cardíaca, descrita na fase indeterminada.

Outro tema pouco avaliado na disautonomia chagásica é a pesquisa de hipotensão ortostática. No estudo ELSA-Brasil, os pacientes com sorologia positiva para DCh apresentavam maior associação com hipotensão ortostática (OR=2,29–IC 95%: 1,2–4,2).^55^ Na verdade, existem discrepâncias nos resultados da avaliação do controle vascular em pacientes chagásicos. Em contraste com outros distúrbios com amplo envolvimento do SNA (por exemplo, DM e amiloidose), na DCh não costuma ser descrita a presença de hipotensão ortostática.^44^^,^^56^

O comprometimento autonômico precoce na DCh sugere que a disautonomia cardiovascular possa estar associada a um aumento da morbimortalidade, arritmias cardíacas e morte súbita.^49^^,^^52^ Ela poderia ser um dos pilares centrais em várias manifestações clínicas, como a disfunção diastólica e/ou sistólica, a dilatação ventricular, as taqui e bradiarritmias e a morte súbita cardíaca.^45^^,^^50^^,^^53^ A disfunção autonômica cardíaca deve ser um fator de risco fisiopatológico determinante ou predisponente na gênese das arritmias. Observa-se maior vulnerabilidade arritmogênica nos casos com disfunções autonômicas mais focais do que nos casos com lesões mais difusas e significativas, em decorrência de maior grau de desconexão do sistema nervoso central, com menor susceptibilidade de interferência do SNA nas propriedades eletrofisiológicas cardíacas.^45^^,^^57^

A observação de taquicardia ventricular sustentada em pacientes com cardiomiopatia chagásica, com função ventricular preservada e denervação simpática miocárdica regional (detectada pela cintilografia com ^123^I-MIBG), bem como a sua ocorrência durante o estresse ortostático em paciente com acometimento discreto da função ventricular e sem alterações eletrocardiográficas basais significativas, favorecem a suposição do papel da disfunção autonômica na fisiopatologia dos distúrbios do ritmo na cardiopatia chagásica.^53^

**Hipotensão Ortostática – Um Sinal Tardio e de Gravidade**

A detecção de hipotensão ortostática neurogênica (HON) representa usualmente uma manifestação tardia e de gravidade, correlacionada com pior prognóstico. Portanto, não se deve aguardar pela sua presença para diagnóstico de disautonomia. Pacientes com patologias conhecidas ou sintomas que comprometam o SNA devem ser investigados precocemente.

## Classificação das Síndromes Clínicas

### Neuropatia Autonômica Cardiovascular

**Figura 2:**
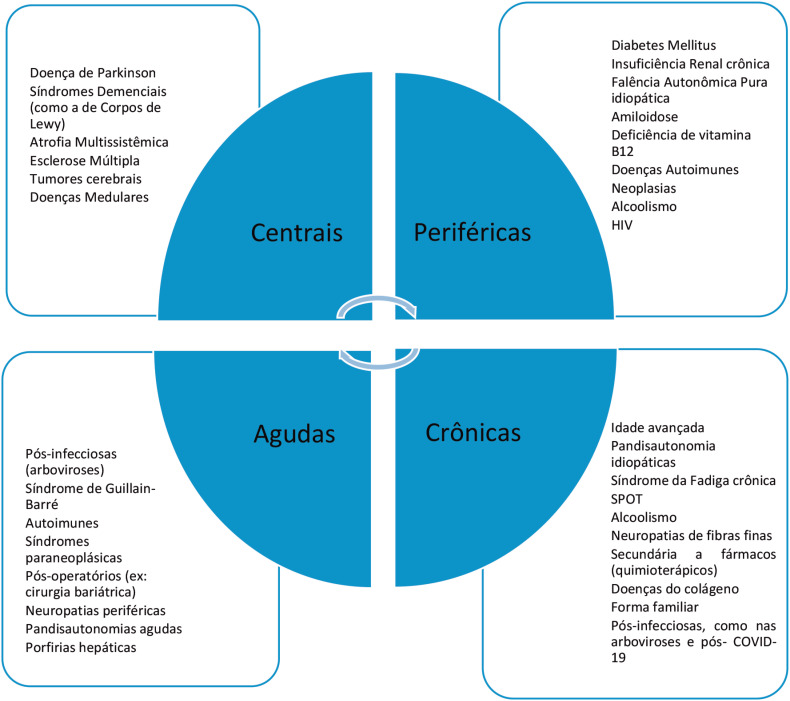
Causas de disautonomia.

Neuropatia Autonômica Cardiovascular (NAC) é um termo muito utilizado pelas Sociedades de Diabetes e Neuropatia Autonômica para expressar o comprometimento do sistema nervoso autônomo cardiovascular na vigência de diabetes mellitus, porém o termo não se restringe a essa patologia.^7^ A NAC engloba aspectos do envolvimento do SNA, desde a fase pré-clínica, que pode ter implicação prognóstica, como na intolerância à glicose ou pré-diabetes. (Figura 2)

Fonte: elaborada pelo próprio autor. A figura exemplifica diversas causas de disfunções autonômicas agudas ou crônicas, centrais ou periféricas. Algumas patologias podem apresentar-se de formas diversas. SPOT: Síndrome Postural Ortostática Taquicardizante.

A expressão hipotensão ortostática neurogênica, muito usada pelos arritmologistas e cardiologistas, vincula a necessidade da presença de HO para definição do diagnóstico, situação que, quando detectada, pode representar uma fase tardia e de maior gravidade, muitas vezes com irreversibilidade do quadro.

### Hipotensão Ortostática Neurogênica (HON) e Hipertensão Supina

A hipotensão ortostática é definida pela presença de redução da pressão arterial (PA) sistólica, de pelo menos 20 mmHg, ou da PA diastólica de 10 mmHg ou ambas, dentro de 3 minutos após posição ortostática ativa ou durante o teste de inclinação.^3^

Nos pacientes que apresentam HON, observa-se comprometimento do sistema nervoso autônomo, caracterizado pela incapacidade de proporcionar adequada vasoconstrição e/ou aumento compensatório adequado da frequência cardíaca (FC), suficientes para manter a PA ao se assumir a posição ortostática. Essa disfunção é atribuída, na maioria dos casos, à liberação insuficiente de norepinefrina a partir dos nervos simpáticos.^42^^,^^43^

Enquanto na HON a vasoconstrição comprometida é decorrente de um dano permanente na atividade eferente simpática, na hipotensão ortostática não neurogênica (HONN), ela inclui uma variedade de causas, como o uso de medicamentos, anti-hipertensivos, antidepressivos e agentes alfa-bloqueadores (tabela 1), além da depleção de volume e doenças crônicas que levam ao descondicionamento físico.^58^

**Tabela 1:** Medicações que podem causar hipotensão ortostática ou exacerbar sintomas de hipotensão ortostática neurogênica

Classe de Medicações	Exemplos
Dopaminérgicos	Levodopa, agonistas da dopamina
Antidepressivos tricíclicos	Amitriptilina, nortriptilina
Anticolinérgicos	Atropina
↓ pré-carga	Furosemida, hidroclorotiazida, espironolactona
Diuréticos	Dinitrato de isossorbida
Inibidores da fosfodiesterase	Sildenafila, vardenafila
Vasodilatadores Antagonistas alfa-1 adrenérgicos Bloqueadores de Ca++ Vasodilatadores diretos	Doxazosina, tansulosina Anlodipino, nifedipina Hidralazina
Inotrópicos e cronotrópicos negativos Betabloqueadores	Propranolol, metoprolol, atenolol, bisoprolol, nebivolol, carvedilol
Bloqueador dos canais de cálcio (não dihidropiridínicos)	Diltiazem, verapamil
Simpaticolíticos de ação central	Clonidina, metildopa
Antagonistas do sistema renina-angiotensina Inibidores da enzima conversora Bloqueadores da angiotensina	Captopril, enalapril, perindopril Losartana, telmisartana, candesartana

Fonte: Adaptada ^4^

É importante diferenciar a HON da HONN devido ao pior prognóstico da HON, com maior morbidade e mortalidade por todas as causas. Além disso, estudos indicam que a presença de HO em indivíduos de meia idade os predispõe a hipertrofia miocárdica mesmo na ausência de hipertensão.^58^^,^^59^ A incidência de HO aumenta com a idade, assim como a hipertensão, a diabetes e as doenças cardiovasculares ou degenerativas.^42^^,^^43^^,^^59^

Os pacientes que apresentam uma das cinco categorias abaixo apresentam maior risco de HON quando comparados com a população geral, e devem ser rotineiramente investigados: w

1. Suspeitos ou diagnosticados com qualquer doença degenerativa associada com disfunção autonômica, incluindo doença de Parkinson, atrofia multissistêmica, insuficiência autonômica pura ou demência por corpos de Lewy;
2. História de quedas inexplicadas ou síncopes;
3. Presença de neuropatia periférica;
4. Idade ≥70 anos com alto grau de fragilidade ou uso de múltiplas medicações;
5. Presença de tonturas ou sintomas ortostáticos inespecíficos.

Após a identificação do paciente com risco de hipotensão ortostática, é importante a realização da medida da PA e da FC em posição supina (após 5 minutos deitado) e no primeiro e terceiro minuto após a posição ortostática, sendo este considerado o padrão ouro para diagnóstico de HO.^58^ Esses valores devem ser aferidos também após 5 minutos de ortostase.

Um método alternativo seria essa aferição após o paciente ter ficado 5 minutos na posição sentada e depois de 3 minutos em posição ortostática. Muitos desses pacientes ainda apresentam hipertensão supina (PA sistólica ≥ 140 mmHg e/ou ≥ a 90 mmHg). Nessa situação, recomenda-se considerar HO se houver queda da PA sistólica ≥30 mmHg e/ou da PA diastólica ≥10 mmHg.^58^

Medidas da FC também variam da posição supina (e/ou sentada) para a ortostática e ajudam a diferenciar a HON da HONN.^42^^,^^59^ Em indivíduos com HO, espera-se uma elevação compensatória da FC de pelo menos 15 bpm dentro de 3 minutos na posição de pé. Se isso não ocorrer, é possível que a HO seja neurogênica (desde que não haja uso concomitante de medicação cronotrópica negativa ou doença do sistema de condução ou paciente seja portador de marca-passo).

Uma revisão dos medicamentos prescritos deve ser realizada com o intuito de se evitar efeitos sobre a resposta barorreflexa (tabela 1), principalmente bloqueadores alfa- e beta-adrenérgicos e agonistas alfa-2 de ação central.

Alguns pacientes podem apresentar hipotensão pós-prandial, particularmente após refeições copiosas, ricas em carboidratos, e associadas com ingestão de bebida alcóolica. Nessas condições, medidas da PA em posição supina e ortostática devem ser realizadas antes e após a refeição, podendo ocorrer usualmente até 90 minutos após a refeição.

Sintomas de intolerância ortostática podem ocorrer em pacientes sem hipotensão ortostática detectável ao exame clínico devido ao comprometimento da vasorreatividade periférica e do retorno venoso. Nesses casos, observa-se um volume sistólico reduzido durante a monitorização hemodinâmica no teste de inclinação ortostática. A resposta compensatória da FC é suficiente para manter a pressão arterial em níveis aceitáveis.^59^^,^^60^

A investigação complementar (tabela 2) é aplicada para desvendar possíveis causas não neurogênicas da HO.^58^

**Tabela 2:** Investigação de pacientes com hipotensão ortostática (HO)

Testes Diagnósticos	
Eletrocardiograma	Avaliar ritmo e distúrbios de condução, hipertrofia, baixa voltagem
Hemograma completo	Avaliar anemia e/ou infecção
Perfil metabólico (sódio, potássio, cálcio, creatinina, ureia, glicemia de jejum, hemoglobina glicada, bicarbonato); Sódio urinário em 24 h	Depleção de volume (relação ureia/creatinina >20 mg/dl; disfunção renal ou diabetes ou distúrbios metabólicos
TSH, T4 livre, Cortisol, ACTH, vitamina B12	Disfunção da tireoide, suprarrenal e deficiência de vitamina B12
Albumina sérica	Desnutrição e doença crônica
Enzimas e função hepática	Em pacientes com perda de peso, suspeita de alcoolismo
Estudo de autoanticorpos (ANNA-1; ANNA-2, Anti-AChr, LGI1, dentre outros) no liquor e/ou sangue	HO de início recente, suspeita de síndrome paraneoplásica autoimune, insuficiência autonômica pura
Eletroforese de proteínas séricas e urinárias, Imunofixação de proteínas Biópsia de nervo, gordura abdominal com coloração vermelho-congo	Em pacientes com neuropatia periférica, suspeita de amiloidose
Catecolaminas plasmáticas em decúbito e após ortostase	Falência autonômica pura
Sorologias para arboviroses (dengue), para COVID-19, Sorologia para HIV	Pesquisas conforme a história clínica
Pesquisa para colagenoses (autoanticorpos como FAN, anti-DNA, anti-SM, anti-RNP)	Suspeita de colagenoses

Fonte: Adaptada;^58^ Anti-AChr: Autoanticorpos antirreceptores gangliônicos de acetilcolina (AChr); ANNA: anticorpo antineuronal nuclear; anti-RNP: anticorpos antirribonucleoproteínas; HIV: vírus da imunodeficiência adquirida. COVID-19: Infecção pelo novo coronavírus tem sido associada a formas de disautonomia como a síndrome da fadiga crônica.

Se as medidas de pressão arterial padronizadas para o diagnóstico de HO não forem eficazes para o diagnóstico, outras condutas podem ser tomadas:

1. Orientar o paciente a medir a PA e a FC em casa em diferentes situações:
  - Quinze minutos depois de se deitar, à noite, ou antes de se levantar pela manhã;
  - Três minutos depois de assumir a posição ortostática, antes de tomar a medicação, ou ainda, sempre que apresentar sintomas;
2. Realizar o teste de inclinação ortostática, que pode documentar uma HO precoce ou tardia;
3. Realizar a monitorização ambulatorial da PA de 24 horas (MAPA) — o paciente deve fazer anotações sempre que se deitar e se levantar.

Quando confirmado o diagnóstico de HO, é importante estabelecer a severidade, que depende da magnitude da queda da PA sistólica, do tempo de tolerância na posição ortostática e da magnitude dos sintomas às atividades da vida diária.

Uma escala de graduação de 1 a 4 (tabela 3) foi proposta como estratificação desses pacientes. Havendo graduação 3 e 4, é aconselhável o encaminhamento para um centro especializado em tratamento da hipotensão ortostática.^61^

**Tabela 3:** Escala de graduação da severidade da hipotensão ortostática neurogênica

Grau	Sintomas e Sinais
1	Sintomas infrequentes/sem restrição para se manter de pé, com 20 a 30 mm Hg de queda da PAS.
2	>30 mmHg de queda na PAS, num tempo de permanência em ortostase ≥5 min
3	>30 mmHg de queda na PAS em um tempo de permanência em ortostase <5 min ou severo impacto na atividade de vida diária
4	>30 mm Hg de queda na PAS em <1 min de permanência em ortostase ou incapacidade funcional.

Fonte: Adaptada.^61^

A hipotensão ortostática pode estar presente em apenas 30–50% dos pacientes com falência autonômica pura e em 60–70 % com atrofia multissistêmica.^33^

## Pandisautonomia e Escores de Avaliação

Diversas patologias podem promover o envolvimento global do SNA, com comprometimento de diversos sistemas e órgãos.

Denomina-se pandisautonomia quando há comprovação de disautonomia sistêmica: cardiovascular e de diversos órgãos. Os pacientes com neuropatia autonômica cardiovascular e/ou hipotensão ortostática neurogênica devem ser questionados sobre sintomatologia específica em outros sistemas.

Alguns questionários podem ser utilizados para melhor avaliação clínica, como o ASP (*Autonomic Symptom Profile*), que contém 73 questões e o COMPASS (*Composite Autonomic Symptom Scale),* que utiliza a escala anterior e quantifica a gravidade das alterações. A validação desses questionários não foi realizada em diversos contextos clínicos. Entretanto, os itens que os compõem podem ser usados como triagem na suspeita de comprometimentos em outros órgãos.^61^^,^^62^

Mais recentemente, um novo escore SAS (*Survey of Autonomic Symptoms)* foi elaborado e validado, mostrando melhor sensibilidade em detectar neuropatias autonômicas leves, não necessitando de métodos complementares e podendo ser uma boa ferramenta clínica para detecção precoce de neuropatia autonômica (Tabela 4).^61^

**Tabela 4:** Questionário SAS (Survey of Autonomic Symptoms) para diagnóstico do envolvimento de diversos órgãos e sistemas nas disautonomias

Sintomas/Problemas de Saúde	Você teve algum destes sintomas nos últimos 6 meses? 1- Sim; 2- Não	Qual a severidade deste sintoma? Escala de 1 a 5 (utilizado se presença de sintomas)
1 - Escurecimento visual	1 ou 2	1–5
2 - Boca Seca ou olhos secos		
3 - Palidez ou cianose		
4 - Sensação de frio em algumas regiões do corpo		
5 - Sudorese dos pés reduzida em relação ao resto do corpo		
6 - Sudorese dos pés reduzida ou ausente após exercícios ou em climas quentes		
7 - Sudorese nas mãos aumentada em relação ao resto do corpo		
8 - Náuseas, vômitos ou gases após alimentações leves		
9 - Diarreia (>3 evacuações por dia)		
10 - Constipação persistente		
11 - Perda de urina		
12 - Dificuldade de ereção		

Fonte: Adaptada.^61^ A presença de três ou mais sintomas conferiu sensibilidade de 95% e especificidade de 65%, enquanto a presença de sete ou mais pontos determinou 60% de sensibilidade e 90% de especificidade. Os sintomas gastrointestinais tiveram menor correlação com outros índices.

**Importância do Diagnóstico Precoce de Disautonomia**

O diagnóstico precoce de Disautonomia, antes das manifestações clínicas ou na presença de sintomas leves, pode trazer implicações terapêuticas e prognósticas importantes.

Em pacientes portadores de diabetes mellitus, o tratamento é mais efetivo com o uso de inibidores do cotransportador de sódio/glicose.

Na amiloidose AF com mutação da transtirretina (ATTR), o Tafamidis e o Inotersen podem modificar a evolução da doença.

A detecção precoce em patologias degenerativas melhora a qualidade de vida e promove redução de quedas, fraturas e internações.

Os testes específicos para avalição de disautonomia possibilitam o diagnóstico antes da sintomatologia incapacitante.

## Síndrome da Fadiga Crônica

Atualmente, é considerada uma doença sistêmica crônica que afeta profundamente a qualidade de vida dos pacientes. Tem sido denominada fadiga crônica ou encefalomielite miálgica devido à documentação de alterações no sistema nervoso central e autônomo. Essa síndrome acomete cerca de 2,5 milhões de indivíduos nos EUA, de todas as idades, e reduz drasticamente a capacidade produtiva.

É uma doença complexa que envolve desregulação do sistema nervoso central, do sistema imune, com disfunção do metabolismo da energia celular e do transporte iônico, além de anormalidades cardiovasculares. É caracterizada por fadiga após o exercício, persistente e recorrente, sem outra causa que explique a origem dos sintomas (Tabela 5).^9^^,^^63^^–^^66^

**Tabela 5:** Critérios Clássicos para Diagnóstico da Síndrome da Fadiga Crônica

Cansaço extremo, persistente ou recorrente, sem causa justificada, com as seguintes características:	
1. Início recente (isto é, não progressivo ao longo da vida) ou com gatilho específico	
2. Dificuldade de exercer atividades habituais (profissionais, físicas ou sociais)	
3. Preencher pelo menos 4 dos seguintes critérios:	
	3.1. Comprometimento da concentração e da memória recente
	3.2. Dor na garganta
	3.3. Linfonodos cervicais ou axilares
	3.4. Dores articulares e musculares
	3.5. Cefaleia
	3.6. Sono não reparador
	3.7. Mal-estar após o exercício, que permanece por período >24 horas.

Fonte: Adaptada^9^

Os exames laboratoriais de rotina geralmente são normais. O acometimento na regulação autonômica do sistema vascular é comumente encontrado, principalmente na resposta deficiente ao assumir a posição ortostática, resultando em alta associação com disautonomia (Figuras 4 e 5).

**Figura 3:**
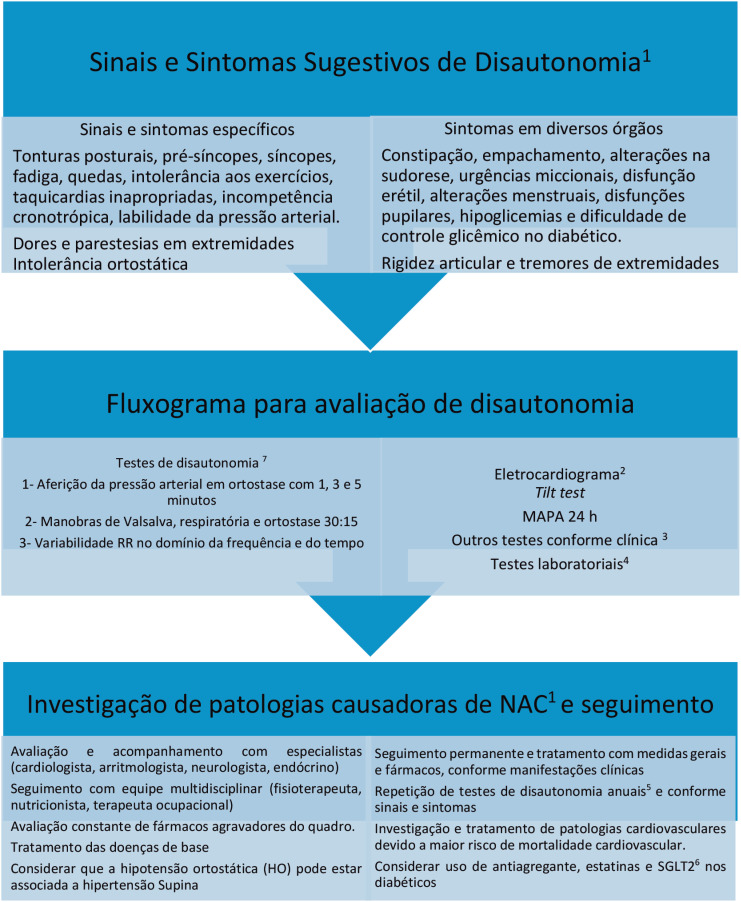
Fluxograma para Avaliação e Seguimento das disautonomias ou neuropatias autonômicas cardiovasculares (NAC) Ocorre de forma idiopática como na atrofia multissistêmica ou na falência autonômica pura, ou em patologias como diabetes mellitus, doenças neurodegenerativas, Doença de Parkinson, síndromes demenciais, insuficiência renal crônica, amiloidose, algumas doenças neoplásicas e nos idosos.Eletrocardiograma para avaliar frequência cardíaca e intervalo QT.Testes neurológicos como eletromiografia, ressonância cerebral, testes cardiológicos como Holter 24 h, avaliação de isquemia.Testes laboratoriais como hemograma completo, função renal, cortisol, ACTH, perfil glicêmico, catecolaminas plasmáticas colhidas deitado e logo após ortostase, marcadores neoplásicos e de doenças autoimunes, dentre outros (vide seção específica).Conforme recomendação das diretrizes internacionais no diabético1,2,7.SGLT2 — Medicamentos para diabetes – inibidores da enzima cotransportadora de sódio-glicose, sendo usados para tratamento da diabetes.Pacientes com extrassístoles muito frequentes, fibrilação atrial, marca-passo cardíaco ou disfunção cognitiva avançada não conseguem ser avaliados por essa metodologia. Considerar que vários fármacos devem ser suspensos para o exame e os valores das medidas devem ser correlacionados com valores de normalidade para a idade e o sexo.

Fonte: elaborada pelo próprio autor.

**Figura 4:**
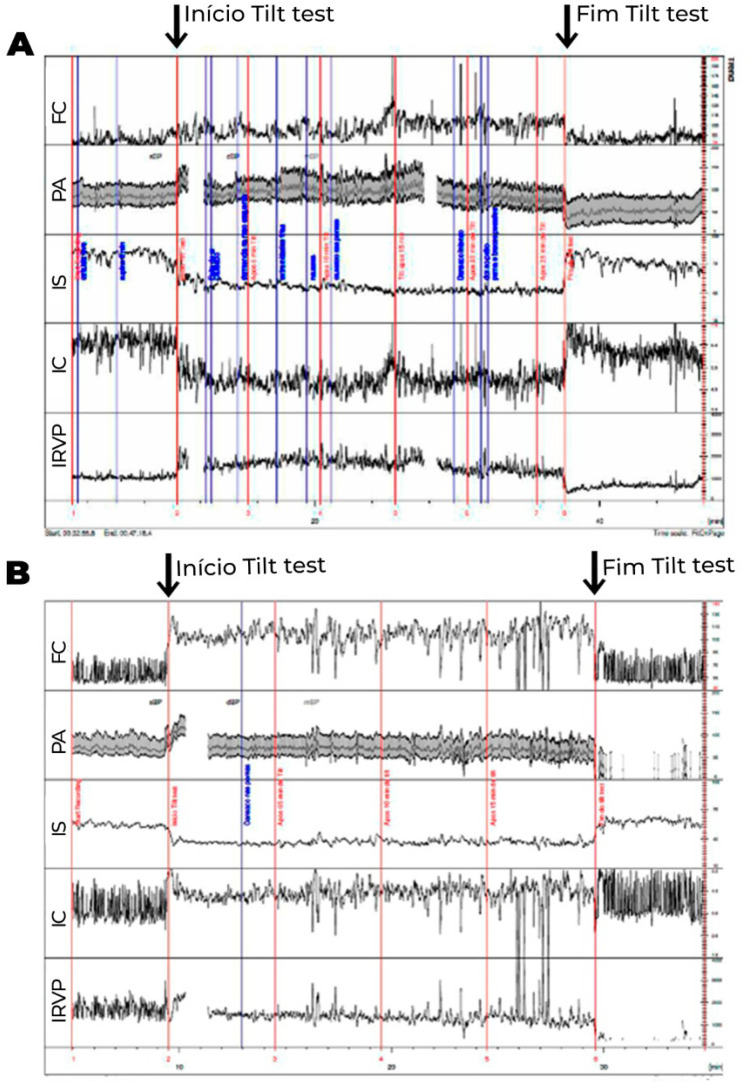
Teste de inclinação com medidas hemodinâmicas, onde os valores do volume sistólico, débito cardíaco e resistência vascular periférica (RVP) foram corrigidos para a superfície corporal, obtendo-se o índice sistólico (IS), índice cardíaco (IC) e índice de RVP (IRVP). 4A. Paciente com diagnóstico clínico de fadiga crônica. Logo após inclinação, observa-se redução exagerada do IS (>30%), compensada inicialmente por elevação esperada do IRVP e da FC. Após 15 minutos de inclinação, observa-se maior redução do IS, associada com redução do IRVP, ao invés de maior elevação compensatória esperada do IRVP, de modo que a compensação para manter a PA estável se faz às custas de maior elevação da FC, que passa a apresentar um aumento exagerado (>30 bpm), do que observado em posição supina. Essa alteração ocorre mais tardiamente (após 10 minutos do início do exame), não preenchendo os critérios para SPOT. 4B. Paciente com diagnóstico de SPOT. Observa-se, durante a inclinação, redução do IS não compensada pela elevação do IRVP. Há redução ao invés de elevação no IRVP ao assumir a posição ortostática. Desse modo, a pressão arterial média (PA) se mantém estável, às custas de elevação exagerada da frequência cardíaca (FC) em >30 bpm ocorrendo nos primeiros 10 minutos de inclinação, associado com sintomas, preenchendo, portanto, critérios para SPOT. A diferença entre as duas entidades pode ser, em alguns casos, apenas temporal.

**Figura 5:**
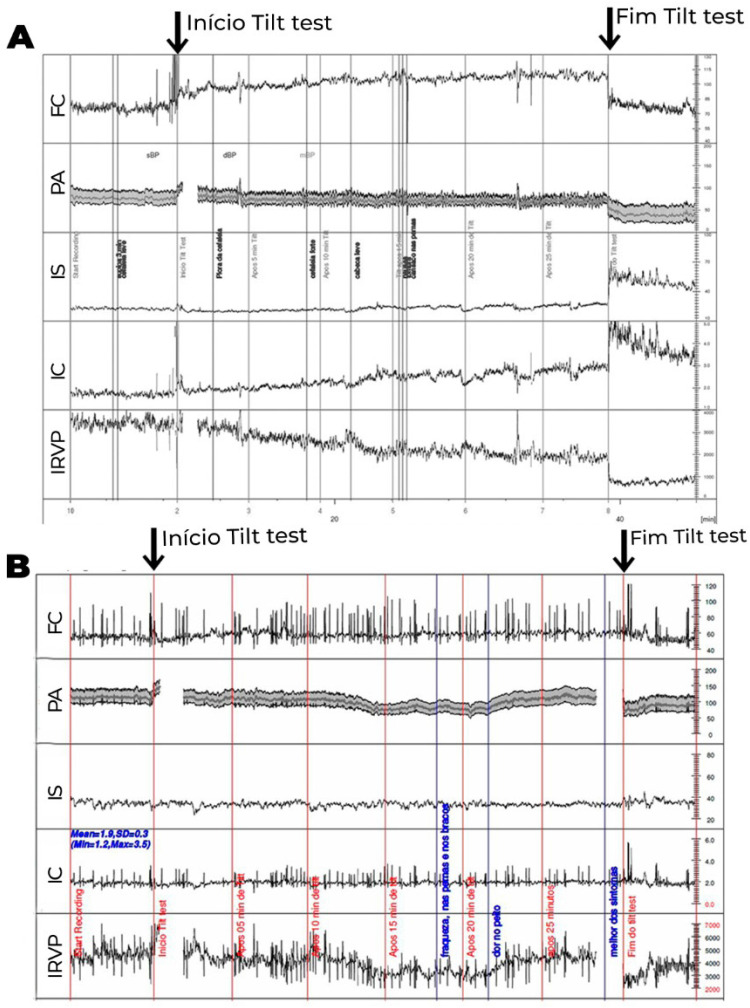
Teste de inclinação com medidas hemodinâmicas, onde volume sistólico, débito cardíaco e resistência vascular periférica (RVP) foram corrigidos para a superfície corporal, obtendo-se índice sistólico (IS), índice cardíaco (IC) e índice de RVP (IRVP). PA: pressão arterial. 5A. Paciente com intolerância ortostática. Não ocorre a elevação esperada do IRVP, que apresenta redução progressiva compensada por elevação progressiva da FC até o final da inclinação, com redução discreta da PA. Os sintomas ocorrem na vigência de déficit na elevação da RVP em posição ortostática. 5B. Paciente com hipotensão ortostática tardia. Neste, também se observa ausência de redução do IS e redução progressiva do IRVP durante inclinação. Após 10 minutos de inclinação, quando ocorre maior redução do IRVP, que não é acompanhada de elevação adicional da FC, observa-se hipotensão ortostática, com sintomas. Nota-se que após 20 minutos ocorre recuperação espontânea do IRVP e da PA, com alívio dos sintomas.

A neuroinflamação pode ter diferentes fatores deflagradores: infecção cerebral (herpes-vírus crônico), autoanticorpos, neurotoxinas ou estresse crônico, e ainda por processos inflamatórios extracerebrais, incluindo o intestino. Baixos níveis de neuroinflamação deflagram alterações comportamentais protetoras, como a redução da atividade, do apetite e aumento do sono.^63^^–^^66^

Exames de ressonância magnética funcional em pacientes com fadiga crônica demonstraram diferentes respostas aos estímulos visual e auditivo e aos testes de memória, assim como alterações de conectividade entre as regiões do cérebro. A tomografia com emissão de pósitrons demonstrou neuroinflamação generalizada e níveis elevados de lactato, que se correlacionam com os graus de fadiga. No líquido espinhal, há maior taxa de proteínas relacionadas com injúria e reparo muscular.^65^^,^^66^

Também têm sido descritas anormalidades metabólicas, que resultam em comprometimento da geração de energia celular de diferentes origens: oxigênio, açúcar, lipídeos e aminoácidos, com altos níveis de estresse oxidativo e de ácido nítrico. Muitos metabólitos encontram-se em níveis inferiores ao normal. Esse quadro hipometabólico é observado em alguns animais em estado de hibernação e permite que animais sob ameaça desacelerem o processo metabólico de consumo energético para preservar as funções vitais.^65^^,^^66^

Entre as anormalidades do sistema nervoso autônomo, observam-se alterações da frequência cardíaca e da pressão arterial durante a posição ortostática prolongada, que não são suficientes para fazer diagnóstico de SPOT, ou de hipotensão ortostática, mas estão associadas com redução do fluxo cerebral e provocam sintomas.

Em testes provocativos de desafios físicos, ortostático e mental, observam-se variados tipos de sintomas, principalmente após 12–24 horas de atividade, conhecidos como “mal-estar após o exercício”. Os pacientes ainda apresentam dificuldade de extração do oxigênio durante o esforço, resultando em limiar anaeróbico reduzido.^67^

Na última década, observou-se um alarmante aumento de pacientes com outras morbidades associadas, como a dor crônica e comprometimento funcional.^46^^,^^47^ Os mesmos critérios diagnósticos podem ser aplicados: fadiga crônica, dor crônica incluindo cefaleia, distúrbios do sono, distúrbios do humor, mal-estar após o exercício, intolerância ortostática e ao exercício e dificuldade de manter a capacidade funcional usual antes do início dos sintomas.

A intolerância ortostática é definida pela presença de tonturas, cabeça leve, turvação visual e pré-síncope, que pioram ao se assumir a posição ortostática e são aliviadas com a postura horizontal.

As doenças crônicas associadas com fadiga crônica, assim como a fadiga crônica isoladamente, tipicamente ocorrem após um evento deflagrador: infecção viral, bacteriana ou fúngica, cirurgia, acidente automobilístico, gravidez, vacinação ou após um período prolongado de estresse físico ou mental. Recentemente, a infecção pelo novo coronavírus (COVID-19) tem demonstrado afetar várias áreas do sistema nervoso, sendo relatados casos suspeitos de fadiga crônica, causando preocupação pela possibilidade de aumento acentuado nessa entidade.^68^^–^^72^

Em alguns casos, nenhum fator precipitante é identificado, mas pode haver história familiar de sintomas semelhantes em membros de primeiro grau, sugerindo um componente genético. Muitos pacientes desenvolvem ansiedade e depressão secundária às doenças crônicas ou como parte das alterações fisiopatológicas da doença de base. Um número significativo de pacientes apresenta marcadores autoimunes e inflamatórios.

Achados objetivos incluem: intolerância ortostática ao teste de inclinação, disfunção autonômica e neuropatia de pequenas fibras (nos testes de função autonômica), hipovolemia e anormalidade em testes funcionais de ressonância magnética (RMN), *Single Photon Emission Computed Tomography* (*SPECT)* ou *Positron Emission Tomography* (*PET* scan). A RMN convencional mostra apenas achados não específicos.^69^^–^^71^

Apesar das recentes descobertas, ainda não há um método de alta sensibilidade e especificidade para o diagnóstico preciso, assim como também não há um tratamento eficaz.

Como parte do tratamento das doenças crônicas associadas com a fadiga crônica, a psicoterapia, a terapia comportamental cognitiva e a terapia ocupacional podem melhorar o estado funcional e reduzir o sofrimento desses pacientes. Geralmente são utilizadas medicações para cefaleia, dor neuropática, tensão muscular, sintomas gastrointestinais e para distúrbios do sono. É de extrema utilidade a separação das diferentes etiologias de fadiga crônica.

A síndrome de ativação dos mastócitos pode causar sintomas de fadiga crônica ou SPOT. Nesse caso, anti-histamínicos podem ser úteis. Nas doenças do tecido conjuntivo, anti-inflamatórios, terapia imunomoduladora como a cloroquina ou imunoglobulina intravenosa e corticoides podem ser utilizados para controle da dor articular e da fadiga.

**Síndrome da Fadiga Crônica – Novos critérios^8^**

Recentemente, recomenda-se que a fadiga crônica seja renomeada como doença sistêmica de intolerância ao exercício, com novos critérios diagnósticos:

1. Fadiga inexplicada e consequente incapacidade para o trabalho por mais que 6 meses;
2. Mal-estar após exercício;
3. Sono não reparador;
4. Comprometimento cognitivo ou intolerância ortostática.

## Síndrome Postural Ortostática Taquicardizante (SPOT)

É definida como a resposta cronotrópica exagerada à mudança da postura horizontal para a ortostase, persistente e associada a sintomas de intolerância ortostática (IO).^73^^,^^74^ É a causa mais comum de IO na população jovem. Afeta de 500.000 a 3.000.000 de indivíduos somente nos EUA, sendo a maioria mulheres (4:1), com idade entre 15 e 25 anos ou no início de sua vida profissional.^10^^,^^11^^,^^75^ Observa-se elevação sustentada da frequência cardíaca (FC) ≥ 30 bpm (≥40 bpm se <20 anos) ou FC ≥120 bpm nos primeiros 10 minutos em posição ortostática ou durante o *tilt test*, sem hipotensão ortostática clássica associada. Pode ocorrer leve redução na pressão arterial.

Geralmente, identifica-se um ou mais fatores desencadeadores: estresse agudo na gravidez, cirurgia, infecção prévia, vacina ou evento traumático. Entre as infecções mais comuns estão: o vírus da mononucleose (18.6%), viroses respiratórias (18%) e gastrointestinais (11.4%).^10^^,^^76^^,^^77^

Na avaliação inicial de pacientes com suspeita de SPOT, além da história e exame físico, os sinais vitais devem ser obtidos em posição supina e ortostática. A história clínica tem como objetivo investigar as possíveis causas de taquicardia ortostática, incluindo os potenciais deflagradores. Os sintomas de SPOT geralmente são exacerbados por exercício, calor, desidratação e ingestão de álcool.

O eletrocardiograma e a monitorização ambulatorial do ECG devem ser realizados para descartar possíveis causas primárias de taquicardias e o ecocardiograma e o teste ergométrico para avaliar a presença de cardiopatia estrutural e a resposta da frequência ao esforço. Testes de função da tireoide, assim como hemograma, devem fazer parte da rotina de investigação para descartar causas secundárias de taquicardia.

O teste de inclinação ortostática pode ser útil para obtenção de parâmetros hemodinâmicos e da tolerância à posição ortostática. A avaliação autonômica ampliada, com análise de vários parâmetros hemodinâmicos durante o teste de inclinação, é altamente recomendável na investigação e diagnóstico diferencial etiológico da SPOT.

Sistemas de monitorização contínuos e não invasivos da PA e ECG, associados a medidas de bioimpedância, permitem a avaliação do volume sistólico, da resistência vascular periférica e do débito cardíaco, sendo possível identificar-se o tipo de distúrbio hemodinâmico presente no paciente com SPOT (Figuras 4 e 5).

A SPOT é uma síndrome heterogênea resultante de distintos mecanismos fisiopatológicos não excludentes. Ela pode ser classificada em cinco tipos, de acordo com o mecanismo fisiopatológico predominante: neuropático, hipovolêmico, hiperadrenérgico. Pode ser secundária a alterações na noradrenalina ou ativação dos mastócitos, e relacionada à hipermobilidade das articulações (síndrome de Ehlers-Danlos).^76^^–^^81^

Na forma neuropática, o principal mecanismo é o comprometimento da vasorreatividade periférica por denervação simpática predominante. Nestes casos, ocorre acúmulo do volume sanguíneo nos membros inferiores ao se assumir a posição ortostática e a ativação do sistema simpático resulta em taquicardia reflexa, nem sempre compensatória. Cerca de 50% desses pacientes apresentam também denervação sudomotora periférica, sugerindo denervação simpática pós-ganglionar.

Na forma hipovolêmica, 70% dos pacientes apresentam hipovolemia decorrente de excessiva retenção de líquidos no compartimento inferior do organismo. Há redução no tônus, aumento da capacitância venosa e redução do volume sistólico durante o teste de inclinação. Essa hipovolemia central resulta em ativação adrenérgica pelos barorreceptores e taquicardia reflexa compensatória exacerbada.

Muitos pacientes desse grupo apresentam volume sanguíneo total reduzido — tanto o volume plasmático quanto de células sanguíneas.^78^^,^^79^ Paradoxalmente, alguns desses pacientes apresentam baixos níveis de atividade da renina e aldosterona plasmática e altos níveis de angiotensina II.^78^

Na forma hiperadrenérgica, a excessiva ativação adrenérgica provoca sintomas como palpitações, sudorese, tremores, ansiedade e até hipertensão desencadeada por atividade física ou estímulo emocional. A forma hiperadrenérgica primária caracteriza-se por altos níveis de norepinefrina plasmática devido a maior produção (1000–2000 pg/ml), ocorrendo em 5 a 10% dos casos.

Na forma secundária, consiste num grupo heterogêneo dividido em três categorias principais:

1. Depuração reduzida de norepinefrina sináptica (mutação de perda da função);
2. Desordem de ativação dos mastócitos caracterizada pela presença de metil-histamina urinária elevada;
3. Bloqueio farmacológico do transporte da norepinefrina por fármacos que inibem esse transporte, como antidepressivos tricíclicos e outras drogas similares à anfetamina, sendo esse último o tipo mais frequentemente encontrado.

Na síndrome de Ehlers-Danlos, uma doença do tecido conjuntivo com hiperelasticidade da pele e hipermotilidade articular, 70% dos indivíduos apresentam SPOT e 18% dos pacientes com SPOT apresentam critérios diagnósticos para síndrome de Ehlers-Danlos, considerado um mecanismo subjacente para a síndrome.^80^

Nos casos de pacientes com SPOT que apresentam a síndrome de ativação dos mastócitos, um fator autoimune pode estar presente. Esses pacientes apresentam rubor da pele e hipertensão associada com a taquicardia ortostática. Ainda não está claro se a ativação simpática causa degranulação dos mastócitos ou se a ativação dos mastócitos causa a vasodilatação.^80^^,^^82^

Em pacientes refratários, uma avaliação extensa em centro especializado em testes autonômicos deve ser considerada. As manobras de Valsalva com medida da PA batimento por batimento podem mostrar uma fase 4 exagerada, revelando excessiva atividade simpática. A dosagem de epinefrina e norepinefrina plasmática em posição supina e ortostática pode ser útil para identificar os casos hiperadrenérgicos, assim como a análise do sódio urinário nas 24 horas e os casos de depleção de volume.^6^

Ansiedade e hipervigilância geralmente são frequentes nos pacientes com SPOT. Entretanto, a elevação da FC não é decorrente do estado de ansiedade, mas decorrente de uma anormalidade fisiológica. Ainda assim, avaliação e acompanhamento psicológicos podem ser úteis no manejo clínico desses pacientes.

Comum a todas as formas de SPOT é o estado de descondicionamento físico. Múltiplos parâmetros associados com o descondicionamento estão presentes nesses pacientes: área e massa cardíacas reduzidas (16%), volume sanguíneo reduzido (20%) e pico do consumo de oxigênio (VO^2^) reduzido quando comparados com controles sedentários. Tanto o repouso no leito quanto o descondicionamento reduzem a sensibilidade do barorreflexo em produzir vasoconstrição.

Num estudo que realizou um registro internacional de SPOT, o condicionamento físico progressivo demonstrou expansão volêmica e aumentou a área cardíaca dos pacientes, resultando numa melhora significativa dos sintomas. Nesse estudo, 71% dos pacientes que completaram o programa de treinamento ficaram livres do diagnóstico de SPOT. Em um pequeno grupo, que foi acompanhado por 6 a 12 meses, o resultado também foi mantido.^83^

O protocolo consistia em oito meses de progressivo treinamento com exercício aeróbico (três sessões por semana), associado com duas sessões semanais de exercício de fortalecimento muscular de baixa resistência, iniciando em posição supina e progredindo para posição ortostática. O exercício, quando comparado com betabloqueador, mostrou melhora na qualidade de vida e normalizou a resposta neuro-humoral, sendo considerado como classe IIa de indicação nas diretrizes internacionais.^11^^,^^83^^,^^84^

Não há nenhuma recomendação classe I para o tratamento da SPOT. Medidas não farmacológicas incluem aumento da ingestão de líquidos para 2 a 3 litros/dia e de sal para 10 a 12 gramas/dia. Infusão de até 2 litros de soro fisiológico é recomendada para descompensações agudas (classe IIb).^11^

**Figura 6:**
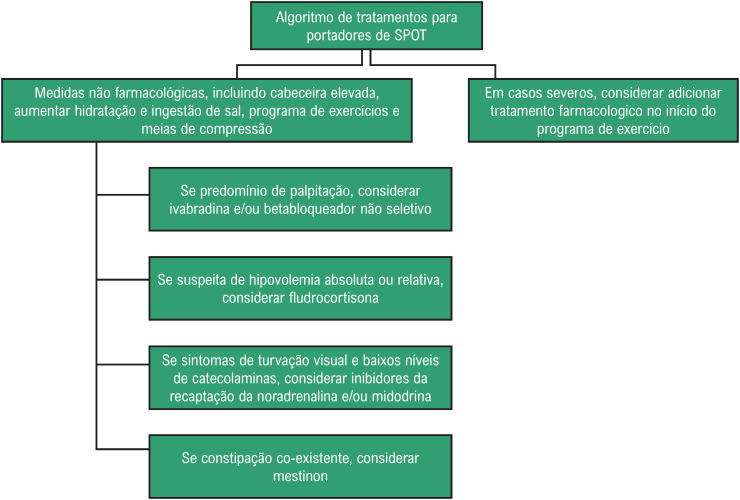
Algoritmo de tratamento para portadores de SPOT.

Se as medidas não farmacológicas não forem efetivas, o tratamento farmacológico pode ser instituído de acordo com o tipo do distúrbio identificado (Figuras 4 e 5) ou o algoritmo proposto por Bryarly et al. modificado (Figura 6).^74^

Fonte: adaptado.^73^

**Síndrome da Fadiga Crônica x Síndrome Postural Ortostática Taquicardizante (SPOT)**

A Síndrome Postural Ortostática Taquicardizante (SPOT) tem sido encontrada em 29% dos pacientes com a síndrome da fadiga crônica, enquanto quase 50% dos pacientes com SPOT apresentam a síndrome da fadiga crônica.

A fludrocortisona pode ser útil na expansão volêmica, mas o seu efeito ainda não foi testado em grandes estudos clínicos. A midodrina, com ação agonista alfa-1 adrenérgico, age aumentando a contração de veias e artérias. Essa medicação significativamente reduz a FC, mas em grau menor que a infusão salina. Apresenta tempo de ação e metabolização rápidos e deve ser utilizada três vezes ao dia, enquanto o paciente estiver em atividade, evitando possível hipertensão noturna.

Medicações como midodrina associadas a baixa dose de betabloqueador não seletivo (propranolol), fludrocortisona e piridostigmina são úteis nas formas disautonômicas e hipovolêmicas da SPOT. Na forma hiperadrenérgica, a clonidina ou alfa-metildopa podem ser eficazes (classe IIb).^11^

A modificação do nó sinusal por radiofrequência não é recomendada e pode ser prejudicial, pois elimina o mecanismo compensatório do baixo débito cerebral, que é a taquicardia sinusal desencadeada pela ação do barorreflexo.

Sintomas concomitantes, como cefaleia e distúrbios do sono ou problemas gastrointestinais são frequentemente observados na SPOT, devendo ser tratados adequadamente, assim como a terapia cognitiva comportamental deve ser considerada.

## Hipersensibilidade do Seio Carotídeo e a Cardioneuroablação

A prevalência da hipersensibilidade do seio carotídeo (HSC) varia com a idade, sendo extremamente incomum em indivíduos com idade <50 anos, apresentando aumento exponencial com a idade. Em pacientes com síncope e idade acima de 60 anos, tem sido observada uma resposta anormal do seio carotídeo em até 22,3%. Portanto, é um achado comum em pacientes idosos sem síncope, especialmente se portadores de doença cardiovascular. Por esta razão, há um consenso de que para o diagnóstico de síndrome da hipersensibilidade do seio carotídeo haja a reprodução dos sintomas clínicos durante a massagem do seio carotídeo e história prévia de síncope espontânea, sugestiva de origem reflexa.^12^^,^^85^^,^^86^ A massagem do seio carotídeo positiva, mas sem história de síncope, define apenas a hipersensibilidade do seio carotídeo e não a síndrome clínica (tabela 6).

**Tabela 6:** Definição de Hipersensibilidade do Seio Carotídeo

DEFINIÇÃO	
Redução da frequência cardíaca e/ou pressão arterial (PA) em resposta à massagem do seio carotídeo:	
	1. Cardioinibitória: pausa ≥3 segundos (usualmente >6 segundos);
	2. Vasodepressora: queda da PA ≥50 mmHg, sem bradicardia significante;
	3. Mista: pausa ≥3 segundos associada com queda da PAS ≥50 mmHg.

Fonte: Adaptada.^1^

A massagem do seio carotídeo é indicação classe I nas diretrizes internacionais para pacientes >40 anos, com síncope de origem desconhecida, compatível com mecanismo reflexo (classe I).^12^ A massagem é entretanto, passível de questionamento, já que pacientes assintomáticos podem apresentar alterações hemodinâmicas com sintomas durante a manobra.^87^ No entanto, se a síncope for de origem indeterminada e a resposta à massagem do seio carotídeo, na forma cardioinibitória, reproduzir o sintoma clínico, existe uma causa presuntiva da síncope, pois o uso de marca-passo nesse grupo de pacientes melhorou os sintomas de síncope em alguns estudos.^11^^,^^88^

Talvez a melhor maneira de confirmar a causa da síncope nesse contexto seria por monitoramento com o ECG de longa duração (*looper* externo ou implantável). Apesar de mais precisa para diagnosticar os casos de hipersensibilidade do seio carotídeo na forma cardioinibitória, essa técnica (*looper* externo ou implantável), não conseguiria identificar as formas vasodepressoras da hipersensibilidade.^89^

A massagem do seio carotídeo deve ser realizada preferencialmente com a monitorização da PA e do eletrocardiograma contínuo, batimento a batimento, sendo mais segura quando realizada no laboratório de teste de inclinação. A manobra deve ser realizada com a face do paciente rodada lateralmente, em posição supina e, se negativa, repetida em posição ortostática, em cada lado, por no máximo 10 segundos de compressão, na região de maior pulsação carotídea, no ângulo formado pela mandíbula, na cartilagem cricoide e na margem anterior do músculo esternocleidomastoideo. Deve-se evitá-la em pacientes com sopro carotídeo antes de uma avaliação adequada.

Embora complicações graves sejam raras (0,24%), o risco de ataque isquêmico transitório deve ser considerado, em especial para pacientes que tenham apresentado tal evento previamente, assim como acidente vascular cerebral, ou que apresentem estenose de artéria carótida >70%, sendo essas as contraindicações para a manobra.^12^

O seio carotídeo é um barorreceptor que responde ao estiramento da parede, como acontece com a elevação da PA.^65^ Nessa situação, ocorre um aumento do tônus vagal e redução do tônus simpático. Do contrário, quando ocorre uma redução da PA e redução da tensão na parede vascular, há uma redução nos disparos do barorreceptor, resultando em atenuação da ação vagal. Os estímulos do barorreflexo são enviados do seio carotídeo para o núcleo do trato solitário, onde fica um grande número de neurônios cardiovasculares.

Embora a fisiologia do barorreflexo do seio carotídeo seja razoavelmente bem compreendida, a fisiopatologia da HSC permanece obscura.

Três principais mecanismos fisiopatológicos têm sido considerados:^90^^–^^94^

Aterosclerose: teoricamente, a redução da complacência dos vasos poderia resultar em redução no fluxo aferente do impulso barorreflexo. Contudo, tem sido demonstrado que a porção aferente do reflexo do seio carotídeo se encontra intacta em indivíduos com HSC.

Denervação do músculo esternocleidomastoideo^92^: com a idade, ocorre denervação do músculo esternocleidomastoideo (demonstrada por eletromiografia), reduzindo assim as informações enviadas para o núcleo do trato solitário, enquanto os barorreceptores do seio carotídeo continuam enviando sinais adequadamente para o mesmo núcleo, gerando um desequilíbrio de informações. Assim, o movimento da cabeça pode resultar em sinais aferentes somente do seio carotídeo, sendo interpretado pelo núcleo do trato solitário como aumento da PA, deflagrando uma redução abrupta da PA e da FC.

Disfunção autonômica generalizada: mais recentemente, tem sido demonstrada atividade simpática elevada em indivíduos com HSC, sintomáticos ou assintomáticos, o que sugere uma disfunção autonômica generalizada.

As manifestações clínicas mais comuns da HSC são síncope, pré-síncope ou tonturas durante manobras com mudança de posição da cabeça. Geralmente, a perda da consciência, assim como sua recuperação, ocorre de forma súbita. Desse modo, injúrias decorrentes das quedas são comumente observadas.

Paciente idosos podem referir episódios como quedas recorrentes, sem causa aparente. Eles podem não referir mudanças de posição da cabeça durante a queda.

Com relação ao tratamento da forma vasodepressora da HSC, estudos com midodrina^95^ e fludrocortisona^96^ demonstraram melhora dos sintomas de síncope e pré-síncope em comparação com placebo. Contudo, para os pacientes com a forma cardioinibitória, o implante de marca-passo definitivo tem sido o tratamento de escolha.

A decisão de se implantar um marca-passo após um único episódio de síncope vai depender da consequência e da severidade da injúria resultante desse episódio. Alguns pequenos estudos observacionais e randomizados mostraram melhora dos sintomas clínicos após o implante.^11^^,^^12^^,^^15^

Entretanto, estudos randomizados cegos, comparando marca-passo dupla câmara versus marca-passo dupla-câmara, sem estimulação ativa (desligados), não mostraram melhora significativa nos pacientes com quedas sem explicação.^88^^,^^90^^,^^97^^,^^98^ Tampouco existem estudos randomizados em larga escala testando o uso do marca-passo na forma cardioinibitória, levantando questionamentos às recomendações das diretrizes vigentes.^97^ Por outro lado, uma metanálise de três estudos demonstrou 9% de recorrência de síncopes em pacientes com estimulação ativa versus 38% no grupo controle (sem marca-passo).^99^ Essa metanálise e outros estudos de revisão são a base de apoio para as recomendações atuais de implante de marca-passo com nível de indicação Classe IIa nas Diretrizes Americanas^15^ e Europeias^11^^,^^15^ de síncope.

A denervação do seio carotídeo por irradiação ou endarterectomia foi considerada no passado também como uma opção de tratamento.^100^

Com relação ao prognóstico, não tem sido observada diferença de mortalidade entre os pacientes com e sem a HSC quando comparados com indivíduos de mesma idade.^87^^,^^101^ Contudo, as consequências de uma injúria decorrente de uma queda num paciente idoso não podem ser adequadamente estimadas. Portanto, os pacientes devem ser informados que o risco de síncope recorrente deve reduzir, mas sintomas menores incluindo pré-síncope podem persistir, mesmo com as terapêuticas implementadas.

Outra estratégia de tratamento muito promissora para a síncope reflexa decorrente de atividade vagal exacerbada é a técnica conhecida como cardioneuroablação, que consiste na modificação da atividade vagal pela técnica de ablação por cateter, utilizando a energia de radiofrequência.^102^

Pachon et al.,^103^ observaram que as fibras nervosas, quando se misturam com as células miocárdicas, produzem alteração na sua condução, de compacta (condução uniforme com uma frequência principal de 40 Hz, que ocorre na região de células muito bem conectadas) para fibrilar (condução com potenciais fracionados com frequência maior que 100 Hz). Os autores utilizaram o padrão de miocárdio fibrilar (encontrado principalmente na região do nó sinusal e do nó atrioventricular) como marcador da interface neuromiocárdio e dos sítios-alvo para cardioneuroablação, obtendo melhora clínica dos episódios de síncope.^103^

Resultados animadores com a técnica de ablação do miocárdio fibrilar ao redor do nó sinusal e do nó atrioventricular têm sido descritos na literatura. Durante o procedimento de ablação, a obtenção de desaparecimento dos potenciais de alta frequência nessas regiões resultou em melhora da função sinusal e nodal.^104^

A cardioneuroablação já foi utilizada para tratar paciente com hipersensibilidade do seio carotídeo, podendo ser uma alternativa ao implante de marca-passo, especialmente em indivíduos jovens que são mais vulneráveis às complicações a longo prazo.^105^^,^^106^

Em resumo, a ablação de plexos ganglionares pode promover significante redução da atividade vagal, nos nós sinusal e atrioventricular, sendo efetiva em reduzir sintomas em pacientes com bradicardia acentuada neuromediada. Devido às diferentes técnicas empregadas, estudos randomizados multicêntricos seriam necessários para definir a eficácia, a melhor técnica, a segurança e a reprodutibilidade do método.^107^

## Taquicardia Sinusal Inapropriada (TSI)

O primeiro caso de taquicardia sinusal inapropriada (TSI) foi descrito na literatura em 1939 por Codvelle e Boucher. ^108^ Atualmente, estima-se uma prevalência de 1,2% na população geral.^11^ É considerada uma condição crônica, mas pouco se sabe sobre sua evolução e mortalidade. Seu mecanismo é pouco compreendido,^109^^–^^113^ incluindo a automaticidade aumentada do nó sinusal, hipersensibilidade beta-adrenérgica, atividade parassimpática reduzida e modulação neuro-hormonal prejudicada.

O início das manifestações geralmente está associado a evento de estresse, tais como um divórcio entre os pais de adolescentes, separação ou outro evento familiar maior. Os sintomas geralmente encontrados são: palpitações, tonturas e síncopes, também podendo ocorrer desconforto abdominal, sudorese, cefaleia, turvação visual, fadiga, ansiedade, intolerância ao exercício, mialgia e dor torácica.

História clínica e exame físico devem ser realizados com o objetivo de identificar possíveis causas para a taquicardia, tais como: hipertireoidismo; medicamentos; uso de substâncias ocultas; deflagradores psicológicos; ataques de pânico e descartar SPOT, considerando que ambas entidades compartilham os mesmos sintomas (Tabela 7).

**Tabela 7:** Causas que devem ser descartadas antes de se fazer o diagnóstico de taquicardia sinusal inapropriada

Condições Médicas		Condições Fisiológicas	Drogas/Substâncias
Hipertireoidismo		Exercício físico	Cafeína
Doença de Cushing		Estresse emocional	Álcool
Feocromocitoma		Dor	Tabaco
Anemia		Febre	Catecolaminas
Infecções		Gravidez	Vasodilatadores
Desidratação		Depleção de volume	Substâncias com atropina
Miocardiopatia			Teofilina
Ataque de Pânico			Drogas ilícitas
Pericardite			Descongestionantes
	Insuficiência mitral ou aórtica		Simpaticomiméticos
Infarto do miocárdio			Hormônios da tireoide
Hipotensão ortostática			

Fonte: próprio autor e adaptada.^10^^,^^110^

Os pacientes devem ser investigados com relação à hipovolemia, que pode ser observada em alguns casos. Entretanto, é necessário se descartar doença cardíaca estrutural para o diagnóstico de TSI. Na história natural de pacientes com TSI, em geral, não há piora da função ventricular decorrente da taquicardia.^109^ Raramente, no entanto, há descrição de casos isolados de taquicardiomiopatia, desafiando a noção de que TSI é sempre condição benigna.^111^^,^^113^^,^^114^

O teste de esforço pode ser útil em documentar uma taquicardia exagerada em resposta ao exercício físico. Os testes autonômicos cardiovasculares, incluindo a resposta da FC tanto à manobra de Valsalva quanto à respiração profunda e à posição ortostática, assim como à variabilidade da FC e à sensibilidade do barorreflexo, não têm demonstrado utilidade clínica, portanto não devem ser rotineiramente empregados.^11^

**Taquicardia Sinusal Inapropriada (TSI)**

É definida quando a frequência cardíaca em repouso é superior a 100 bpm e a média da FC é maior que 90 bpm no Holter 24 h em adolescentes e adultos jovens. Ocorre mais comumente em mulheres, sem causa que justifique. Está associada a diversos sintomas acentuados e às vezes debilitantes, sendo principalmente palpitações, tonturas e síncopes.

Os portadores de TSI geralmente apresentam uma perda significativa da qualidade de vida. Não existem estudos clínicos prospectivos, controlados com placebo, para as intervenções terapêuticas utilizadas no tratamento, podendo alguns sintomas persistirem apesar do controle da FC.

Existem algumas evidências de que a ivabradina, na dose de 5 a 7,5 mg, duas vezes ao dia, possa melhorar a qualidade de vida. ^115^^,^^116^^,^^117^ Além disso, parece que a ivabradina pode trazer benefícios quando associada com o betabloqueador (metoprolol).^118^

Betabloqueadores, isoladamente, não são úteis e podem causar efeitos colaterais. Outros tratamentos têm sido propostos, tais como: fármacos como a fludrocortisona; clonidina; eritropoietina; medidas não farmacológicas, como as meias de compressão elásticas; os exercícios físicos e, raramente, a ablação por radiofrequência, que pode trazer riscos de lesão do nó sinusal, necessitando de implante de marca-passo cardíaco.^119^ Os pacientes portadores de TSI geralmente requerem atenção especial e mudanças no estilo de vida.

## Observação

A parte II desse artigo, com a descrição das manifestações clínicas, cardiovasculares, métodos de investigação e tratamento, continuará nos próximos números da revista.

## References

[1] 1. Spallone V, Ziegler D, Freeman R, Bernardi L, Frontoni S, Pop-Busui R, et al. Cardiovascular autonomic neuropathy in diabetes: clinical impact, assessment, diagnosis, and management. Diabetes Metab Res Rev. 2011 Oct;27(7):639–53. 10.1002/dmrr.1239 21695768

[2] 2. Spallone V. Update on the impact, diagnosis and management of cardiovascular autonomic neuropathy in diabetes: what is defined, what is new, and what is unmet. Diabetes Metab J. 2019 Feb;43(1):3–30. 10.4093/dmj.2018.0259 PMC6387879 30793549

[3] 3. Gibbons CH, Schmidt P, Biaggioni I, Frazier-Mills C, Freeman R, Isaacson S, et al. The recommendations of a consensus panel for the screening, diagnosis, and treatment of neurogenic orthostatic hypotension and associated supine hypertension. J Neurol. 2017;264(8):1567–82. 10.1007/s00415-016-8375-x PMC5533816 28050656

[4] 4. Jordan J, Fanciulli A, Tank J, Calandra-Buonaura G, Cheshire WP, Cortelli P, et al. Management of supine hypertension in patients with neurogenic orthostatic hypotension: scientific statement of the American Autonomic Society, European Federation of Autonomic Societies, and the European Society of Hypertension. J Hypertens. 2019 Aug;37(8):1541–6. 10.1097/HJH.0000000000002078 30882602

[5] 5. Vinik AI, Camacho PM, Davidson JA, Handelsman Y, Lando HM, Leddy AL, et al. American Association of Clinical Endocrinologists and American College of Endocrinology Position Statement on Testing for Autonomic and Somatic Nerve Dysfunction. Endocr Pract. 2017 Dec;23(12):1472–8. 10.4158/EP-2017-0053 29320641

[6] 6. Spallone V, Bellavere F, Scionti L, Maule S, Quadri R, Bax G, et al. Recommendations for the use of cardiovascular tests in diagnosing diabetic autonomic neuropathy. Nutr Metab Cardiovasc Dis. 2011;21(1):69–78. 10.1016/j.numecd.2010.07.005 21247746

[7] 7. Freeman R, Abuzinadah AR, Gibbons C, Jones P, Miglis MG, Sinn DI. Orthostatic hypotension. J Am Coll Cardiol. 2018;72(11):1294–309. 10.1016/j.jacc.2018.05.079 30190008

[8] 8. Institute of Medicine, Board on the Health of Select Populations, Committee on the Diagnostic Criteria for Myalgic Encephalomyelitis/Chronic Fatigue Syndrome. Beyond Myalgic Encephalomyelitis/Chronic Fatigue Syndrome: Redefining an Illness. Washington (DC): National Academies Press; 2015. 25695122

[9] 9. Fukuda K, Straus SE, Hickie I, Sharpe MC, Dobbins JG, Komaroff A. The chronic fatigue syndrome: a comprehensive approach to its definition and study. International Chronic Fatigue Syndrome Study Group. Ann Intern Med.1994;121(12):953-9. 10.7326/0003-4819-121-12-199412150-00009 7978722

[10] 10. Sheldon RS, Grubb 2nd BP, Olshansky B, Shen WK, Calkins H, Brignole M, et al. 2015 heart rhythm society expert consensus statement on the diagnosis and treatment of postural tachycardia syndrome, inappropriate sinus tachycardia, and vasovagal syncope. Heart Rhythm. 2015 Jun;12(6):e41–63. 10.1016/j.hrthm.2015.03.029 PMC5267948 25980576

[11] 11. Brignole M, Moya A, Lange FJ, Deharo JC, Elliott PM, Fanciulli A, et al. 2018 ESC Guidelines for the diagnosis and management of syncope. 10.5603/KP.2018.0161 30117520

[12] 12. Brignole M, Moya A, Lange FJ, Deharo JC, Elliott PM, Fanciulli A, et al. Practical Instructions for the 2018 ESC Guidelines for the diagnosis and management of syncope. Eur Heart J. 2018 Jun 1;39(21):e43–80. 10.1093/eurheartj/ehy071 29562291

[13] 13. Gondim FAA, Barreira AA, Claudino R, Cruz MW, Cunha FMB, Freitas MRG, et al. Definition and diagnosis of small fiber neuropathy: consensus from the Peripheral Neuropathy Scientific Department of the Brazilian Academy of Neurology. Arq Neuro-Psiquiatr. 2018;76(3):200–8. 10.1590/0004-282x20180015 29809227

[14] 14. Tesfaye S, Boulton AJM, Dyck PJ, Freeman R, Horowitz M, Kempler P, et al. Diabetic neuropathies: update on definitions, diagnostic criteria, estimation of severity, and treatments. Diabetes Care. 2010;33(10):2285–93. 10.2337/dc10-1303 PMC2945176 20876709

[15] 15. Shen WK, Sheldon RS, Benditt DG, Cohen MI, Forman DE, Goldberger ZD, et al. 2017 ACC/AHA/HRS Guideline for the Evaluation and Management of Patients With Syncope: A Report of the American College of Cardiology/American Heart Association Task Force on Clinical Practice Guidelines and the Heart Rhythm Society. J Am Coll Cardiol.2017;Aug 1;70(5):620-63. 10.1016/j.jacc.2017.03.003 28286221

[16] 16. Bernardi L, Spallone V, Stevens M, Hilsted J, Frontoni S, Pop-Busui R, et al. Methods of investigation for cardiac autonomic dysfunction in human research studies. Diabetes Metab Res Rev. 2011;27(7):654–64. 10.1002/dmrr.1224 21695761

[17] 17. Shibao C, Lipsitz LA, Biaggioni I. ASH position paper: evaluation and treatment of orthostatic hypotension. J Clin Hypertens. 2013;15(3):147–53. 10.1111/jch.12062 PMC8033893 23458585

[18] 18. Ricci F, De Caterina R, Fedorowski A. Orthostatic Hypotension: Epidemiology, Prognosis, and Treatment. J Am Coll Cardiol. 2015 Aug 18;66(7):848–60. 10.1016/j.jacc.2015.06.1084 26271068

[19] 19. Freeman R, Wieling W, Axelrod FB, Benditt DG, Benarroch E, Biaggioni I, et al. Consensus statement on the definition of orthostatic hypotension, neurally mediated syncope and the postural tachycardia syndrome. Clin Auton Res. 2011;21(2):69-72. 10.1007/s10286-011-0119-5 21431947

[20] 20. Langley JN. The autonomic nervous system. Cambridge: W. Heffer & Sons; 1921.

[21] 21. Hasan W. Autonomic cardiac innervation. Organogenesis. 2013;9(3):176–93. 10.4161/org.24892 PMC3896589 23872607

[22] 22. Jose AD, Collison D. The normal range and determinants of the intrinsic heart rate in man. Cardiovasc Res. 1970;4(2):160–7. 10.1093/cvr/4.2.160 4192616

[23] 23. Opthof T. The normal range and determinants of the intrinsic heart rate in man. Cardiovascular Research. 2000;45(1):177–84. 10.1016/s0008-6363(99)00322-3 10841627

[24] 24. Schreihofer AM, Guyenet PG. The baroreflex and beyond: control of sympathetic vasomotor tone by GABAergic neurons in the ventrolateral medulla. Clin Exp Pharmacol Physiol. 2002;29(5):514–21. 10.1046/j.1440-1681.2002.03665.x 12010201

[25] 25. Smit AA, Halliwill JR, Low PA, Wieling W. Pathophysiological basis of orthostatic hypotension in autonomic failure. J Physiol. 1999 Aug 15;519(Pt 1):1–10. 10.1111/j.1469-7793.1999.0001o.x PMC2269496 10432334

[26] 26. Ponte CMM, Fernandes VO, Gurgel MHC, Vasconcelos ITGF, Karbage LBAS, Liberato CBR, et al. Early commitment of cardiovascular autonomic modulation in Brazilian patients with congenital generalized lipodystrophy. BMC Cardiovasc Disord. 2018 Jan 12;18(1):6. 10.1186/s12872-017-0738-4 PMC5767058 29329523

[27] 27. Rolim LCSP, Sá JR, Chacra AR, Dib SA. Diabetic cardiovascular autonomic neuropathy: risk factors, clinical impact and early diagnosis. Arq Bras Cardiol. 2008 Apr;90(4):e24–31. 10.1590/s0066-782x2008000400014 18516377

[28] 28. Pop-Busui R, Evans GW, Gerstein HC, Fonseca V, Fleg JL, Hoogwerf BJ, et al. Effects of cardiac autonomic dysfunction on mortality risk in the action to control cardiovascular risk in diabetes (ACCORD) trial. Diabetes Care. 2010;33(7):1578–84. 10.2337/dc10-0125 PMC2890362 20215456

[29] 29. Maser RE, Mitchell BD, Vinik AI, Freeman R. The association between cardiovascular autonomic neuropathy and mortality in individuals with diabetes: a meta-analysis. Diabetes Care. 2003 Jun;26(6):1895–901. 10.2337/diacare.26.6.1895 12766130

[30] 30. Vinik AI, Casellini C, Parson HK, Colberg SR, Nevoret ML. Cardiac autonomic neuropathy in diabetes: a predictor of cardiometabolic events. Front Neurosci. 2018 Aug 27;12:591. 10.3389/fnins.2018.00591 PMC6119724 30210276

[31] 31. Low PA, Vernino S, Suarez G. Autonomic dysfunction in peripheral nerve disease. Muscle Nerve. 2003 Jun;27(6):646–61. 10.1002/mus.10333 12766975

[32] 32. Shy GM, Drager GA. A neurological syndrome associated with orthostatic hypotension: a clinical-pathologic study. Arch Neurol. 1960 May;2:511–27 10.1001/archneur.1960.03840110025004 14446364

[33] 33. Committee on the Diagnostic Criteria for Myalgic Encephalomyelitis/Chronic Fatigue Syndrome. Beyond myalgic encephalomyelitis/chronic fatigue syndrome: redefining an illness. Washington (DC): National Academies Press; 2015. V. 46. 25695122

[34] 34. Vernino S, Low PA, Fealey RD, Stewart JD, Farrugia G, Lennon VA. Autoantibodies to ganglionic acetylcholine receptors in autoimmune autonomic neuropathies. N Engl J Med. 2000 Sep 21;343(12):847–55. 10.1056/NEJM200009213431204 10995864

[35] 35. Low PA. Laboratory evaluation of autonomic function. In: Clinical Autonomie Disorders, 2nd ed. Philadelphia: Lippincott-Raven; 1997.

[36] 36. Andrade C. A peculiar form of peripheral neuropathy; familiar atypical generalized amyloidosis with special involvement of the peripheral nerves. Brain. 1952 Sep;75(3):408–27. 10.1093/brain/75.3.408 12978172

[37] 37. Corino de Andrade. BMJ. 2005;331:163.

[38] 38. Bittencourt PL, Couto CA, Clemente C, Farias AQ, Palacios SA, Mies S, et al. Phenotypic expression of familial amyloid polyneuropathy in Brazil. Eur J Neurol. 2005;12(4):289–93. 10.1111/j.1468-1331.2004.00941.x 15804246

[39] 39. Kittleson MM, Maurer MS, Ambardekar AV, Bullock-Palmer RP, Chang PP, Eisen HJ, et al. Cardiac amyloidosis: evolving diagnosis and management: a scientific statement from the American Heart Association. Circulation. 2020;142(1):e7-22. 10.1161/CIR.0000000000000792 32476490

[40] 40. Berk J, Damy T, Drachman B, Elliott P, Gottlieb S, Grogan M,et all. Efficacy of tafamidis in transthyretin amyloid cardiomyopathy in the ATTR-ACT trial. Heart Lung. 2019;48(5):470.

[41] 41. Ruzieh M, Dasa O, Pacenta A, Karabin B, Grubb B. Droxidopa in the treatment of postural orthostatic tachycardia syndrome. Am J Ther. 2017;24(2):e157–61. 10.1097/MJT.0000000000000468 27563801

[42] 42. Loavenbruck A, Sandroni P. Neurogenic orthostatic hypotension: roles of norepinephrine deficiency in its causes, its treatment, and future research directions. Curr Med Res Opin. 2015;31(11):2095–104. 10.1185/03007995.2015.1087988 26373628

[43] 43. Ruzieh M, Batizy L, Dasa O, Oostra C, Grubb B. The role of autoantibodies in the syndromes of orthostatic intolerance: a systematic review. Scand Cardiovasc J. 2017 Oct;51(5):243–7. 10.1080/14017431.2017.1355068 28738696

[44] 44. Amorim DS, Neto JAM. Functional alterations of the autonomic nervous system in Chagas’ heart disease. Sao Paulo Med J. 1995;113(2):772–84. 10.1590/s1516-31801995000200007 8650476

[45] 45. Junqueira Jr LF. Insights into the clinical and functional significance of cardiac autonomic dysfunction in Chagas disease. Rev Soc Bras Med Trop. 2012 Mar;45(2):243–52. 10.1590/s0037-86822012000200020 22535000

[46] 46. Dávila DF, Inglessis G, Dávila CAM. Chagas’ heart disease and the autonomic nervous system. Int J Cardiol. 1998;66:123–7. 10.1016/s0167-5273(98)00212-5 9829322

[47] 47. Chagas C, Villela E. Cardiac form of American Trypanosomiasis. Mem Inst Oswaldo Cruz. 1922;14(1):5–61.

[48] 48. Goin JC, Borda E, Leiros CP, Storino R, Sterin-Borda L. Identification of antibodies with muscarinic cholinergic activity in human Chagas’ disease: pathological implications. J Auton Nerv Syst. 1994 Apr;47(1-2):45–52. 10.1016/0165-1838(94)90064-7 8188983

[49] 49. Ribeiro ALP, Moraes RS, Ribeiro JP, Ferlin EL, Torres RM, Oliveira E, et al. Parasympathetic dysautonomia precedes left ventricular systolic dysfunction in Chagas disease. Am Heart J. 2001;141(2):260–5. 10.1067/mhj.2001.111406 11174350

[50] 50. Punukollu G, Gowda RM, Khan IA, Navarro VS, Vasavada BC. Clinical aspects of the Chagas’ heart disease. Int J Cardiol. 2007;115(3):279–83. 10.1016/j.ijcard.2006.03.004 16769134

[51] 51. Ribeiro ALP, Campos MS, Baptista LMG, Sousa MR. The Valsalva maneuver in Chagas disease patients without cardiopathy. Clin Auton Res. 2010;20(2):79–83. 10.1007/s10286-009-0044-z 19941031

[52] 52. Marino VSP, Dumont SM, Mota LG, Braga DS, Freitas SS, Moreira MCV. Sympathetic dysautonomia in heart failure by 123I-MIBG: comparison between Chagasic, non-Chagasic and heart transplant patients. Arq Bras Cardiol. 2018 Aug;111(2):182–90. 10.5935/abc.20180124 PMC6122917 30088556

[53] 53. Nunes MCP, Dones W, Morillo CA, Encina JJ, Ribeiro AL, Council on Chagas Disease of the Interamerican Society of Cardiology. Chagas disease: an overview of clinical and epidemiological aspects. J Am Coll Cardiol. 2013 Aug 27;62(9):767–76. 10.1016/j.jacc.2013.05.046 23770163

[54] 54. Sterin-Borda L, Borda E. Role of neurotransmitter autoantibodies in the pathogenesis of chagasic peripheral dysautonomia. Ann N Y Acad Sci. 2000;917:273–80. 10.1111/j.1749-6632.2000.tb05393.x 11270349

[55] 55. Velten APC, Benseñor I, Souza JB, Mill JG. Factors associated with orthostatic hypotension in adults: the ELSA-Brasil study. Cad Saúde Pública. 2019;35(8):e00123718. 10.1590/0102-311X00123718 31411271

[56] 56. Marin-Neto JA. Cardiac dysautonomia and pathogenesis of Chagas’ heart disease. Int J Cardiol. 1998 Sep 30;66(2):129–31. 10.1016/s0167-5273(98)00213-7 9829323

[57] 57. Elias Neto J, Kuniyoshi RR, Silva MA, Merçon E. Taquicardia ventricular polimórfica durante teste de inclinação na doença de Chagas. J Card Arrhythm. 2017;30(2):51–4.

[58] 58. Fanciulli A, Jordan J, Biaggioni I, Calandra-Buonaura G, Cheshire WP, Cortelli P, et al. Consensus statement on the definition of neurogenic supine hypertension in cardiovascular autonomic failure by the American Autonomic Society (AAS) and the European Federation of Autonomic Societies (EFAS) : Endorsed by the European Academy of Neurology (EAN) and the European Society of Hypertension (ESH). Clin Auton Res. 2018 Aug;28(4):355–62. 10.1007/s10286-018-0529-8 PMC6097730 29766366

[59] 59. Távora-Mehta MZP, Mehta N, Magajevski A, Oliveira L, Maluf DLS, Concato L, et al. Reduced systolic volume: main pathophysiological mechanism in patients with orthostatic intolerance? Arq Bras Cardiol. 2016;107(4):354-64. 10.5935/abc.20160135 PMC5102482 27849259

[60] 60. Low PA. Neurogenic orthostatic hypotension: pathophysiology and diagnosis. Am J Manag Care.2015;21(13 Suppl):s248–57. 26790109

[61] 61. Zilliox L, Peltier AC, Wren PA, Anderson A, Smith AG, Singleton JR, et al. Assessing autonomic dysfunction in early diabetic neuropathy: the survey of autonomic symptoms. Neurology. 2011;76(12):1099–105. 10.1212/WNL.0b013e3182120147 PMC3068012 21422460

[62] 62. Low PA. Composite autonomic scoring scale for laboratory quantification of generalized autonomic failure. Mayo Clin Proc. 1993 Aug;68(8):748–52. 10.1016/s0025-6196(12)60631-4 8392653

[63] 63. Lewis I, Pairman J, Spickett G, Newton JL. Clinical characteristics of a novel subgroup of chronic fatigue syndrome patients with postural orthostatic tachycardia syndrome. J Intern Med. 2013;273(5): 501–10. 10.1111/joim.12022 23206180

[64] 64. Carruthers BM, Sande MI, De Meirleir KL, Klimas NG, Broderick G, Mitchell T, et al. Myalgic encephalomyelitis: International Consensus Criteria. J Intern Med. 2011;270(4):327–38. 10.1111/j.1365-2796.2011.02428.x PMC3427890 21777306

[65] 65. Mueller C, Lin JC, Sheriff S, Maudsley AA, Younger JW. Evidence of widespread metabolite abnormalities in Myalgic encephalomyelitis/chronic fatigue syndrome: assessment with whole-brain magnetic resonance spectroscopy. Brain Imaging Behav. 2020;14(2):562–72. 10.1007/s11682-018-0029-4 PMC6612467 30617782

[66] 66. Campen CLMC, Rowe PC, Visser FC. Blood volume status in ME/CFS correlates with the presence or absence of orthostatic symptoms: preliminary results. Front Pediatr. 2018 Nov 15;6:352. 10.3389/fped.2018.00352 PMC6262290 30525014

[67] 67. Stevens S, Snell C, Stevens J, Keller B, VanNess JM. cardiopulmonary exercise test methodology for assessing exertion intolerance in myalgic encephalomyelitis/chronic fatigue syndrome. Front Pediatr. 2018 Sep 4;6:242. 10.3389/fped.2018.00242 PMC6131594 30234078

[68] 68. Solve M.E. What does COVID-19 portend for ME/CFS; 2020. [acesso 30 jan 2021]. Disponível em: https://solvecfs.org/covid/.

[69] 69. Blitshteyn S, Chopra P. Chronic fatigue syndrome: from chronic fatigue to more specific syndromes. Eur Neurol. 2018;80(1-2):73–7. 10.1159/000493531 30286454

[70] 70. Sotzny F, Blanco J, Capelli E, Castro-Marrero J, Steiner S, Murovska M, et al. Myalgic encephalomyelitis/chronic fatigue syndrome – evidence for an autoimmune disease. Autoimmun Rev. 2018;17(6):601–9. 10.1016/j.autrev.2018.01.009 29635081

[71] 71. Komaroff A, Cho TA. Role of infection and neurologic dysfunction in chronic fatigue syndrome. Semin Neurol. 2011;31(3):325–37. 10.1055/s-0031-1287654 21964849

[72] 72. Komaroff AL. Advances in understanding the pathophysiology of chronic fatigue syndrome. JAMA. 2019;322(6):499-500.73. 10.1001/jama.2019.8312 31276153

[73] 73. Bryarly M, Phillips LT, Fu Q, Vernino S, Levine BD. Postural orthostatic tachycardia syndrome: JACC Focus Seminar. J Am Coll Cardiol. 2019 Mar 19;73(10):1207–28. 10.1016/j.jacc.2018.11.059 30871704

[74] 74. Thieben MJ, Sandroni P, Sletten DM, Benrud-Larson LM, Fealey RD, Vernino S, et al. Postural orthostatic tachycardia syndrome: the Mayo clinic experience. Mayo Clin Proc. 2007;82(3):308–13. 10.4065/82.3.308 17352367

[75] 75. Zadourian A, Doherty TA, Swiatkiewicz I, Taub PR. Postural orthostatic tachycardia syndrome: prevalence, pathophysiology, and management. Drugs. 2018 Jul;78(10):983–94. 10.1007/s40265-018-0931-5 29943373

[76] 76. Boris JR, Bernadzikowski T. Demographics of a large paediatric Postural Orthostatic Tachycardia Syndrome Program. Cardiol Young. 2018;28(5):668–74. 10.1017/S1047951117002888 29357955

[77] 77. Levin KH, Chauvel P.. Clinical neurophysiology: diseases and disorders. Amsterdam: Elsevier BV; 2019. (Handbook of clinical neurology 3rd series; vol. 161).

[78] 78. Raj SR, Biaggioni I, Yamhure PC, Black BK, Paranjape SY, Byrne DW, et al. Renin-aldosterone paradox and perturbed blood volume regulation underlying postural tachycardia syndrome. Circulation. 2005;111(13):1574–82. 10.1161/01.CIR.0000160356.97313.5D 15781744

[79] 79. Fu Q, VanGundy TB, Melyn Galbreath M, Shibata S, Jain M, Hastings JL, et al. Cardiac origins of the postural orthostatic tachycardia syndrome. J Am Coll Cardiol. 2010;55(25):2858–68. 10.1016/j.jacc.2010.02.043 PMC2914315 20579544

[80] 80. Wallman D, Weinberg J, Hohler AD. Ehlers-Danlos Syndrome and Postural Tachycardia Syndrome: a relationship study. J Neurol Sci. 2014 May 15;340(1-2):99–102. 10.1016/j.jns.2014.03.002 24685354

[81] 81. Garland EM, Celedonio JE, Raj SR. Postural Tachycardia Syndrome: beyond orthostatic intolerance. Curr Neurol Neurosci Rep. 2015 Sep;15(9):60. 10.1007/s11910-015-0583-8 PMC4664448 26198889

[82] 82. Shibao C, Arzubiaga C, Jackson Roberts L, Raj S, Black B, Harris P, et al. Hyperadrenergic Postural Tachycardia Syndrome in mast cell activation disorders. Hypertension. 2005;45(3):385–90. 10.1161/01.HYP.0000158259.68614.40 15710782

[83] 83. George SA, Bivens TB, Howden EJ, Saleem Y, Melyn Galbreath M, Hendrickson D, et al. The international POTS registry: evaluating the efficacy of an exercise training intervention in a community setting. Heart Rhythm. 2016;13(4):943–50. 10.1016/j.hrthm.2015.12.012 26690066

[84] 84. Fu Q, Vangundy TB, Shibata S, Auchus RJ, Williams GH, Levine BD. Exercise training versus propranolol in the treatment of the postural orthostatic tachycardia syndrome. Hypertension. 2011 Aug;58(2):167–75. 10.1161/HYPERTENSIONAHA.111.172262 PMC3142863 21690484

[85] 85. Krediet CTP, Parry SW, Jardine DL, Benditt DG, Brignole M, Wieling W. The history of diagnosing carotid sinus hypersensitivity: why are the current criteria too sensitive? Europace. 2011;13(1):14–22. 10.1093/europace/euq409 21088002

[86] 86. Kerr SRJ, Pearce MS, Brayne C, Davis RJ, Kenny RA. Carotid sinus hypersensitivity in asymptomatic older persons. Arch Intern Med. 2006;166(5):515-20. 10.1001/archinte.166.5.515 16534037

[87] 87. Wu TC, Hachul DT, Darrieux FCC, Scanavacca MI. Carotid sinus massage in syncope evaluation: a nonspecific and dubious diagnostic method. Arq Bras Cardiol. 2018;111(1):84-91. 10.5935/abc.20180114 PMC6078367 30110049

[88] 88. Parry SW, Steen N, Bexton RS, Tynan M, Kenny RA. Pacing in elderly recurrent fallers with carotid sinus hypersensitivity: a randomised, double-blind, placebo controlled crossover trial. Heart. 2009;95:405–9. 10.1136/hrt.2008.153189 19124530

[89] 89. Elias Neto J, Vasconcelos DM, Merçon ES, Silva MA< Kuniyoshi R. Ablação do splexos gangliônicos parassimpáticos cardíacos no tratamento da síncope neuromediada cardioinibitória em paciente com monitor de evento implantável. Arquivos Brasileiros de Cardiologia.2018;111(5):S1.

[90] 90. Amin V, Pavri BB. Carotid sinus syndrome. Cardiol Rev. 2015;23(3):130–4. 10.1097/CRD.0000000000000041 25211534

[91] 91. Kenny RA, Lyon CC, Ingram AM, Bayliss J, Lightman SL, Sutton R. Enhanced vagal activity and normal arginine vasopressin response in carotid sinus syndrome: implications for a central abnormality in carotid sinus hypersensitivity. Cardiovasc Res. 1987;21(7):545–50. 10.1093/cvr/21.7.545 3677144

[92] 92. Blanc J-J, L’Heveder G, Mansourati J, Tea SH, Guillo P, Mabin D. Assessment of a newly recognized association. Carotid sinus hypersensitivity and denervation of sternocleidomastoid muscles. Circulation. 1997;95(11):2548–51. 10.1161/01.cir.95.11.2548 9184585

[93] 93. Tan MP, Kenny RAM, Chadwick TJ, Kerr SRJ, Parry SW. Carotid sinus hypersensitivity: disease state or clinical sign of ageing? Insights from a controlled study of autonomic function in symptomatic and asymptomatic subjects. Europace. 2010 Nov;12(11):1630–6. 10.1093/europace/euq317 20823040

[94] 94. Kumar NP, Thomas A, Mudd P, Morris RO, Masud T. The usefulness of carotid sinus massage in different patient groups. Age Ageing. 2003;32(6):666–9. 10.1093/ageing/afg114 14600010

[95] 95. Moore A, Watts M, Sheehy T, Hartnett A, Clinch D, Lyons D. Treatment of vasodepressor carotid sinus syndrome with midodrine: a randomized, controlled pilot study. J Am Geriatr Soc. 2005;53(1):114–8. 10.1111/j.1532-5415.2005.53021.x 15667387

[96] 96. Costa D, McIntosh S, Kenny RA. Benefits of fludrocortisone in the treatment of symptomatic vasodepressor carotid sinus syndrome. Br Heart J. 1993;69(4):308–10. 10.1136/hrt.69.4.308 PMC1025042 8489861

[97] 97. Parry SW. Should we ever pace for carotid sinus syndrome? Front Cardiovasc Med. 2020;7(44):1-11. 10.3389/fcvm.2020.00044 PMC7188762 32391383

[98] 98. Ryan DJ, Nick S, Colette SM, Roseanne K. Carotid sinus syndrome, should we pace? A multicentre, randomised control trial (Safepace 2). Heart. 2010;96(5):347-51. 10.1136/hrt.2009.176206 19933747

[99] 99. Brignole M, Menozi C. The natural history of carotid sinus syncope and the effect of cardiac pacing. Europace.2011;13:462-4. 10.1093/europace/euq516 21447520

[100] 100. Trout 3rd HH, Brown LL, Thompson JE. Carotid sinus syndrome. Ann Surg. 1979;189(5): 575–80. 10.1097/00000658-197905000-00006 PMC1397187 443910

[101] 101. Brignole M, Oddone D, Cogorno S, Menozzi C, Gianfranchi L, Bertulla A. Long-term outcome in symptomatic carotid sinus hypersensitivity. Am Heart J. 1992;123(3):687–92. 10.1016/0002-8703(92)90507-r 1539520

[102] 102. Pachon JC, Pachon EI, Pachon JC, Lobo TJ, Pachon MZ, Vargas RNA, et al. “Cardioneuroablation” – new treatment for neurocardiogenic syncope, functional AV block and sinus dysfunction using catheter RF-ablation. Europace. 2005;7(1):1–13. 10.1016/j.eupc.2004.10.003 15670960

[103] 103. Pachon-M JC. Neurocardiogenic syncope: Pacemaker or cardioneuroablation? Heart Rhythm. 2020;17(5 PtA):829–30. 10.1016/j.hrthm.2020.02.026 32113895

[104] 104. Lu CS, Guo CJ, Fang DP, Hao P, He D-F, Xu AG. Initial experience with ablation of the innervation surrounding sinus and atrioventricular nodes to treat paroxysmal bradyarrhythmia. Chin Med J. 2020 Jan 20;133(2):134–40. 10.1097/CM9.0000000000000595 PMC7028169 31880742

[105] 105. Palamà Z, De Ruvo E, Grieco D, Borrelli A, Sciarra L, Calò L. Carotid sinus hypersensitivity syncope: is there a possible alternative approach to pacemaker implantation in young patients? Postepy Kardiol Interwencyjnej. 2017;13(2):184–5. 10.5114/pwki.2017.67993 PMC5545653 28798796

[106] 106. Pachon M JC, Pachon M EI, Lobo TJ, Pachon M JC, Pachon MZC, Vargas RNA, et al. Syncopal high-degree AV block treated with catheter RF ablation without pacemaker implantation. Pacing Clin Electrophysiol. 2006;29(3):318–22. 10.1111/j.1540-8159.2006.00340.x 16606401

[107] 107. Scanavacca M, Hachul D. Ganglionated plexi ablation to treat patients with refractory neurally mediated syncope and severe vagal-induced bradycardia. Arq Bras Cardiol. 2019;112(6):709-12. 10.5935/abc.20190107 PMC6636377 31314822

[108] 108. Codvelle MM, Boucher H. Tachycardie sinusale permanente à haute fréquence sans troubles fonctionnels. Bull Mem Soc Med Hop Paris. 1939;54:1849–52.

[109] 109. Olshansky B, Sullivan RM. Inappropriate sinus tachycardia. J Am Coll Cardiol. 2013;61(8):793–801. 10.1016/j.jacc.2012.07.074 23265330

[110] 110. Chiale PA, Garro HA, Schmidberg J, Sánchez RA, Acunzo RS, Lago M, et al. Inappropriate sinus tachycardia may be related to an immunologic disorder involving cardiac β andrenergic receptors. Heart Rhythm. 2006;3(10):1182–6. 10.1016/j.hrthm.2006.06.011 17018348

[111] 111. Peyrol M, Lévy S. Clinical presentation of inappropriate sinus tachycardia and differential diagnosis. J Interv Card Electrophysiol. 2016 Jun;46(1):33–41. 10.1007/s10840-015-0051-z 26329720

[112] 112. Winum PF, Cayla G, Rubini M, Beck L, Messner-Pellenc P. A case of cardiomyopathy induced by inappropriate sinus tachycardia and cured by ivabradine. Pacing Clin Electrophysiol. 2009;32(7):942–4. 10.1111/j.1540-8159.2009.02414.x 19572874

[113] 113. Morillo CA, Klein GJ, Thakur RK, Li H, Zardini M, Yee R. Mechanism of “inappropriate” sinus tachycardia. Role of sympathovagal balance. Circulation. 1994;90(2):873–7. 10.1161/01.cir.90.2.873 7913886

[114] 114. Sag S, Coskun H, Baran I, Güllülü S, Aydinlar A. Inappropriate sinus tachycardia-induced cardiomyopathy during pregnancy and successful treatment with ivabradine. Anatol J Cardiol. 2016;16(3):212-13. 10.14744/AnatolJCardiol.2016.6813 PMC5336809 27067557

[115] 115. Shabtaie SA, Witt CM, Asirvatham SJ. Natural history and clinical outcomes of inappropriate sinus tachycardia. J Cardiovasc Electrophysiol. 2020 Jan;31(1):137–43. 10.1111/jce.14288 31749258

[116] 116. Cappato R, Castelvecchio S, Ricci C, Bianco E, Vitali-Serdoz L, Gnecchi-Ruscone T, et al. Clinical efficacy of ivabradine in patients with inappropriate sinus tachycardia: a prospective, randomized, placebo-controlled, double-blind, crossover evaluation. J Am Coll Cardiol. 2012;60(15):1323–9. 10.1016/j.jacc.2012.06.031 22981555

[117] 117. Ptaszynski P, Kaczmarek K, Cygankiewicz I, Klingenheben T, Urbanek I, Wranicz JK. Ivabradine in patients with synptomatic inapprioprate sinus tachycardia:long-term observational study. J Am Coll Cardiol. 2017;69(11):306.

[118] 118. Ptaszynski P, Kaczmarek K, Ruta J, Klingenheben T, Cygankiewicz I, Wranicz JK. Ivabradine in combination with metoprolol succinate in the treatment of inappropriate sinus tachycardia. J Cardiovasc Pharmacol Ther. 2013;18(4):338–44. 10.1177/1074248413478172 23426376

[119] 119. Marrouche NF, Beheiry S, Tomassoni G, Cole C, Bash D, Dresing T, et al. Three-dimensional nonfluoroscopic mapping and ablation of inappropriate sinus tachycardia. Procedural strategies and long-term outcome. J Am Coll Cardiol. 2002;39(6):1046–54. 10.1016/s0735-1097(02)01703-5 11897449

