# Supplementary material for: Disautonomia: Uma Condição Esquecida – Parte 1
Source: Arq Bras Cardiol. 2021 Apr 8;116(4):814–35. [Article in Portuguese] doi: 10.36660/abc.20200420 (PMC8121406; doi:10.36660/abc.20200420)
Supplement: Translation

# Translation: Dysautonomia: A Forgotten Condition — Part 1

**Authors:** Eduardo Arrais Rocha, Niraj Mehta, Maria Zildany Pinheiro Távora-Mehta, Camila Ferreira Roncari, Alan Alves de Lima Cidrão, Jorge Elias

**Affiliations:** 1 FortalezaCE Brazil Hospital Universitário Walter Cantídio da Universidade Federal do Ceará (UFC) - Programa de Pós-graduação em Ciências Cardiovasculares da Faculdade de Medicina da UFC, Fortaleza, CE - Brazil; 2 CuritibaPR Brazil Universidade Federal do Paraná, Curitiba, PR - Brazil; 3 CuritibaPR Brazil Clínica de Eletrofisiologia do Paraná, Curitiba, PR - Brazil; 4 FortalezaCE Brazil Departamento de Fisiologia e Farmacologia - Faculdade de Medicina da Universidade Federal do Ceará (UFC), Fortaleza, CE - Brazil; 5 FortalezaCE Brazil Programa de Pós-graduação em Ciências Cardiovasculares da Faculdade de Medicina da UFC, Fortaleza, CE - Brazil; 6 VitóriaES Brazil Serviço de Eletrofisiologia do Vitória Apart Hospital, Vitória, ES - Brazil

**Keywords:** Dysautonomia, Syncope, Hypotension Orthostatic, Chronic Fatigue Disease, Amyloidosis, Chagas Disease, COVID-19, Cardiovascular Autonomic Neuropathy, Carotid Sinus Hipersensitivity, Diabetes mellitus

## Abstract

Dysautonomia covers a range of clinical conditions with different characteristics and prognoses. They are classified as Reflex Syndromes, Postural Orthostatic Tachycardia Syndrome (POTS), Chronic Fatigue Syndrome, Neurogenic Orthostatic Hypotension (nOH) and Carotid Sinus Hypersensitivity Syndrome. Reflex (vasovagal) syndromes will not be discussed in this article.

1. Reflex (vasovagal) syndromes are mostly benign and usually occur in patients without an intrinsic autonomic nervous system (ANS) or heart disease. Therefore, they are usually studied separately.
2. Cardiovascular Autonomic Neuropathy (CAN) is the term most currently used to define dysautonomia with impairment of the sympathetic and/or parasympathetic cardiovascular autonomic nervous system. It can be idiopathic, such as multisystemic atrophy or pure autonomic failure, or secondary to systemic pathologies such as diabetes mellitus, neurodegenerative diseases, Parkinson's disease, dementia syndromes, chronic renal failure, amyloidosis and it may also occur in the elderly.
3. The presence of Cardiovascular Autonomic Neuropathy (CAN) implies greater severity and worse prognosis in various clinical situations.
4. Detection of Orthostatic Hypotension (OH) is a late sign and means greater severity in the context of dysautonomia, defined as Neurogenic Orthostatic Hypotension (nOH). It must be differentiated from hypotension due to hypovolemia or medications, called non-neurogenic orthostatic hypotension (nnOH).
5. OH can result from benign causes, such as acute, chronic hypovolemia or use of various drugs. However, these drugs may only reveal subclinical pictures of Dysautonomia. All drugs of patients with dysautonomic conditions should be reevaluated.
6. Precise diagnosis of CAN and the investigation of the involvement of other organs or systems is extremely important in the clinical suspicion of pandysautonomia.
7. In diabetics, in addition to age and time of disease, other factors are associated with a higher incidence of CAN, such poor glycemic control, hypertension, dyslipidemia and obesity. Among diabetic patients, 38–44% can develop Dysautonomia, with prognostic implications and higher cardiovascular mortality. In the initial stages of DM, autonomic dysfunction involves the parasympathetic system, then the sympathetic system and, later on, it presents as orthostatic hypotension.
8. Valsalva, Respiratory and Orthostatic tests (30:15) are the gold standard methods for the diagnosis of CAN. They can be associated with RR Variability tests in the time domain, and mainly in the frequency domain, to increase the sensitivity (protocol of the 7 tests). These tests can detect initial or subclinical abnormalities and assess severity and prognosis.
9. The Tilt Test should not be the test of choice for investigating CAN at an early stage, as it detects cases at more advanced stages. Tilt response with a dysautonomic pattern (gradual drop in blood pressure without increasing heart rate) may suggest CAN.
10. Treatment of patients at moderate to advanced stages of dysautonomia is quite complex and often refractory, requiring specialized and multidisciplinary evaluation. There is no cure for most types of Dysautonomia at a late stage.
11. NOH patients can progress with supine hypertension in more than 50% of the cases, representing a major therapeutic challenge. The immediate risk and consequences of OH should take precedence over the later risks of supine hypertension and values greater than 160/90 mmHg are tolerable. Sleeping with the head elevated (20–30 cm), not getting up at night, taking short-acting antihypertensive drugs for more severe cases, such as losartan, captopril, clonidine or nitrate patches, may be necessary and effective in some cases.
12. Preventive measures such as postural care; good hydration; higher salt intake; use of compression stockings and abdominal straps; portioned meals; supervised physical activity, mainly sitting, lying down or exercising in the water are important treatment steps.
13. Various drugs can be used for symptomatic nOH, especially fludrocortisone, midodrine and droxidopa, the latter not available in Brazil. The risk of exacerbation or triggering supine hypertension should be considered.
14. Chronic Fatigue Syndrome represents a form of Dysautonomia and has been renamed as a systemic disease of exercise intolerance, with new diagnostic criteria: 1 - Unexplained fatigue, leading to occupational disability for more than 6 months; 2 - Feeling ill after exercising; 3 - Non-restorative sleep; 4 - One of the following findings: cognitive impairment or orthostatic intolerance. Several pathologies today have evolved with chronic fatigue, being called chronic diseases associated with chronic fatigue.
15. Postural orthostatic tachycardia syndrome (POTS), another form of presentation of dysautonomic syndromes, is characterized by sustained elevation of heart rate (HR) ≥30 bpm (≥40 bpm if <20 years) or HR ≥120 bpm, in the first 10 minutes in an orthostatic position or during the tilt test, without classical orthostatic hypotension associated. A slight decrease in blood pressure may occur. Symptoms appear or get worse in an orthostatic position, with dizziness, weakness, pre-syncope, palpitations, and other systemic symptoms being common.

**Vasovagal Syndromes x Dysautonomia**

Vasovagal syndromes are clinical situations that are different from cardiovascular autonomic neuropathies, as they do not represent intrinsic diseases in the Autonomic Nervous System (ANS), resulting from reflex, transient, benign mechanisms, therefore having a favorable prognosis.

## Dysautonomia: A frequent and underdiagnosed condition

The autonomic nervous system (ANS) regulates important functions in various organic systems such as cardiovascular, digestive, genital-urinary and sudomotor systems. Its dysfunctions can determine several clinical manifestations, some of which are debilitating and serious. Various pathologies can compromise the ANS and determine symptoms, increasing the risk of syncope, falls and higher cardiovascular mortality. Due to the different clinical manifestations and the poor familiarity of professionals, Dysautonomia is often underdiagnosed, being recognized at more advanced stages, with debilitating and incapacitating symptoms and worse prognosis.

The term cardiovascular autonomic neuropathy (CAN) means involvement of the autonomic nervous system, related to cardiovascular functions. Diabetes mellitus (DM) represents the most common and studied form of CAN and serves as a model for understanding and investigating several other pathologies.^1^^,^^2^

In the diabetic population, it is known as Diabetic Cardiovascular Autonomic Neuropathy, with a prevalence of 20% in patients with DM, up to 54% in type 1 (DM1) and 46% in type 2 (DM2), between 40 and 70 years. In diabetics, in addition to age and time of disease, other factors are associated with a greater risk of CAN, such as poor glycemic control, hypertension, dyslipidemia and obesity. In the initial stages of DM, autonomic dysfunction involves the parasympathetic system, then the sympathetic system and, later, it evolves to orthostatic hypotension.

The cardiovascular autonomic nervous system modulates heart rate, diastolic and systolic volumes, QT interval and systemic vascular resistance. Its impairment is related to increased cardiovascular morbidity and mortality.

The purpose of this review is to provide relevant information on the different forms of autonomic dysfunctions, their clinical manifestations, diagnostic and therapeutic methodologies, and prognostic implications. We emphasize the importance of diagnosis, of its distinction with vasovagal reflex syndromes and the need for greater dissemination of information on these pathologies, since it is little remembered in general clinical practice. Reflex vasovagal syndromes will not be addressed in this chapter.

Various guidelines were considered in this review, including: Cardiovascular Autonomic Neuropathy (CAN) Guidelines, Consensus Statement on Neurogenic Orthostatic Hypotension and Supine Hypertension, Syncope Guidelines, Guidelines on CAN in Diabetics, Guidelines on Cardiovascular Tests in Autonomic Neuropathy, Consensus Statement on the Investigation of Autonomic Dysfunction in Human Research Studies, Consensus Statement on the Diagnosis and Treatment of Postural Orthostatic Tachycardia Syndrome and Inappropriate Sinus Tachycardia, and other studies. Discussions between specialists of the Brazilian Society of Cardiac Arrhythmias were included, considering the lack of major studies on various topics covered in this study.^1^^–^^20^

## Physiology of the Autonomic Nervous System

The autonomic nervous system (ANS) plays an important role in the control of visceral functions through the sympathetic and parasympathetic subdivisions.

The ANS provides neurovegetative adjustments for the expression of motivated behaviors or compensatory responses to internal and external stimuli in order to promote the maintenance of homeostasis, along with the endocrine system. The term “autonomic nervous system” was proposed by Langley, in 1898, as the nomenclature used until then had different connotations and were inaccurate as to the recently discovered functions of this system.^20^

For easier comprehension the ANS is commonly analyzed for its anatomical, neurochemical and functional aspects. The basic organization involves two neuronal groups arranged in series and connected by a chemical synapse. The second neuron in this series is completely outside the central nervous system and its cellular body is located in the autonomic ganglia, from where axonal projections come out, which will innervate the target organs; hence their denomination as postganglionic neurons.^21^

The neurons that send axonal which send axonal projections from the central nervous system to the ganglia, making synapse with the cellular bodies present in these structures are called preganglionic neurons.

The anatomical difference between sympathetic and parasympathetic ANS concerns the location of the cellular bodies of preganglionic neurons. Sympathetic preganglionic neurons are located in thoracic and lumbar segments of the spinal cord and the parasympathetic ones are located in the brain stem and in the sacral segments of the spinal cord.

Regarding neurochemistry, all preganglionic neurons are cholinergic and use acetylcholine as a neurotransmitter. Despite some exceptions, parasympathetic postganglionic neurons release acetylcholine in the target organ, while sympathetic postganglionic neurons release noradrenaline.

The adrenal medullary cells are homologous to the sympathetic postganglionic neurons nd primarily secrete adrenaline and, to a lesser extent, norepinephrine directly into the bloodstream, in response to stimulation by sympathetic preganglionic neurons.

Finally, the sympathetic and parasympathetic nervous systems differ as to the responses triggered in the target organs. A few structures receive single innervation, while most organs receive double innervation. The responses induced by sympathetic and parasympathetic ANS stimulation can be antagonistic or cooperative.

As shown in figure 1, systemic blood vessels are innervated by sympathetic ANS. Greater activation of α^1^-adrenergic receptors through increased sympathetic tone or adrenaline release by the adrenal gland causes vasoconstriction in most systemic blood vessels, especially in the vessels of the abdominal viscera, an important vascular resistance bed with great influence on the determination of blood pressure (BP).

**Figure 1:** Schematic representation of heart and blood vessels innervation by the sympathetic and parasympathetic ANS. Parasympathetic neurons are represented in red and sympathetic neurons are represented in green. SA node — sinoatrial node; AV node — atrioventricular node. For better viewing, a single schematic spinal segment was represented and the images are not represented on the same graphic scale.

In contrast, reduced sympathetic tone or plasma levels of adrenaline results in vasodilation. Coronary blood vessels particularly express β^2^ receptors and undergo vasodilation in response to adrenaline.

The heart is innervated by the sympathetic and parasympathetic systems (Figure 1). Cardiac parasympathetic innervation is directed to the sinoatrial (SA) and atrioventricular (AV) nodes and acetylcholine binds to the M2 muscarinic acetylcholine receptors expressed in the nodal cells, inducing a negative chronotropic effect. On the other hand, sympathetic ANS innervates both the SA and AV nodes, as well as the ventricular muscle. Noradrenaline induces positive chronotropic and inotropic effects by acting on β^1^-adrenergic receptors.^22^

All cardiac cells, in principle, have the electrical property of automatism; however, under physiological conditions, SA nodal cells present spontaneous depolarization in a higher frequency and take control of the heartbeat, and are thus considered the cardiac pacemaker.

Upon pharmacological blockade of muscarinic and β-adrenergic receptors, the intrinsic heart rate generated by the sinoatrial node is approximately 100 beats per minute, suggesting that there is a predominance of parasympathetic influence on the heart.^23^ For BP adjustments, the sympathetic and parasympathetic tone for the heart and blood vessels are often modified by the baroreflex.

Blood pressure (BP) is constantly monitored by high-pressure baroreceptors (stretch receptors) found in the aortic arch and carotid sinus, which send signaling through the vagus and glossopharyngeal nerve, respectively, to the nucleus of the solitary tract (NTS), located in the dorsomedial portion of the medulla.^24^

When BP is high, the baroreceptors are more activated and, by baroreflex mechanisms, there is an increase in parasympathetic tone and a reduction in sympathetic tone to the heart and blood vessels. Increased baroreceptor firing rate activates the NTS, which in turn activates the nucleus ambiguus (NA), the bulbar nucleus where the parasympathetic preganglionic neurons cellular bodies are located, resulting in an increase in parasympathetic tone. In parallel, the NTS also activates the caudal ventrolateral medulla (CVLM), which sends inhibitory projections to the rostral ventrolateral medulla (RVLM). RVLM neurons are considered pre-sympathetic, because they project into the spinal cord intermediolateral cell column and synapse with the cellular bodies of sympathetic preganglionic neurons. Therefore, the greater activity of CVLM results in inhibition of RVLM and, consequently, reduction of sympathetic tone.

On the other hand, the lower activity of baroreceptors when BP is decreased results in: 1) less NA activation and, therefore, reduction of parasympathetic tone; and 2) less CVLM activation and, consequently, greater RVLM activity and increased sympathetic tone to the heart and blood vessels.

Changes in the normal functioning of the baroreflex mechanism can trigger pathological conditions called dysautonomia, such as neurogenic orthostatic hypotension, for example. The change from the supine to the orthostatic position increases the gravitational resistance to venous return, resulting in decreased end diastolic volume and, consequently, systolic volume (SV), observed in several pathologies.

BP is directly proportional to total peripheral resistance and cardiac output, the latter being the volume of blood pumped by the heart per minute, that is, SV multiplied by heart rate (HR).

Thus, reduced SV on switching to the orthostatic position induces hypotension. In healthy individuals, this hypotension is transient as the baroreflex mechanisms are quickly activated and cause an increase in contractile force and HR and systemic vasoconstriction, compensatory responses that normalize BP. In individuals with dysautonomia, prolonged hypotension called neurogenic orthostatic hypotension (nOH), may occur.

**Multiple system atrophy (MSA) — Shy-Dragger syndrome**

The complete syndrome consists of orthostatic hypotension, bladder and bowel incontinence, loss of sweating, iris atrophy, external eye paralysis, stiffness, tremors, loss of movement, impotence, fasciculations, distal muscle atrophy and evidence of neuropathic lesions. The onset is usually is the 5th–7th decade of life.

## Pathophysiology and clinical presentations

Various pathophysiological mechanisms have been described in autonomic nervous system (ANS) abnormalities. They may vary depending on specific etiologies, such as diabetes or amyloidosis. Several situations, however, have their causal mechanisms unknown.

Although other neurotransmitters are important in the regulation of cardiovascular responses, the release of noradrenaline in sympathetic postganglionic nerve endings is the most important mediator of the rapid cardiovascular regulation required in blood pressure balance and cerebral perfusion. Neurogenic orthostatic hypotension represents a deficiency in the responsiveness of this neurotransmitter to postural change.

Unlike reflex or vasovagal syndromes, in dysautonomia conditions, the reflexes of increased heart rate preceding the clinical picture and bradycardia concomitant with hypotension are not observed.

In diabetes mellitus, metabolic and vascular abnormalities occur that can justify neurological damage. Hyperglycemia, accumulation of sorbitol, fructose and end products of advanced glycation, with bindings to receptors in the smooth endothelial and muscle cells of vasa nervorum Schwann cells and macrophages may contribute to neurological damage. Oxidative stress leading to depletion of antioxidant cellular enzymes and activation of inflammation cascade, with deterioration of cellular organelles, especially at the mitochondrial level, are other mechanisms that culminate in vascular occlusion, endothelial dysfunction and neuroinflammation, determining toxicity and neuronal death.^25^^–^^30^

Sinucleinopathy, a condition that involves Parkinson's Disease, Lewy body dementia, pure autonomic failure (Bradbury and Eggleston syndrome) and multiple system atrophy (Shy and Dragger syndrome), causes intracellular deposition and aggregation of a protein called alpha-synuclein in different areas of the central and peripheral nervous system.^19^^,^^31^^,^^32^

Multiple system atrophy (MSA),^32^ a more severe and rare idiopathic form, described in 1960, comes in two forms: 1. Parkinsonism: muscle stiffness and bradykinesia are observed (it is different from the classical Parkinson's disease, in which tremors prevail) 2. Cerebellar MSA: ataxia symptoms. Both forms have involvement of the autonomic nervous system.^8^ Nuclear magnetic resonance imaging of the brain reveal cerebellar, pons or peduncle atrophy, or hypersignal on the pons, known as the hot cross bun sign, which may occur later. Catecholamine dosages are usually normal, as it is a preganglionic autonomic polyneuropathy.

In pure autonomic failure, of idiopathic etiology, described in 1925 and known as postganglionic autonomic polyneuropathy, the symptoms are gradual, progressive, and may involve severe and debilitating conditions, with severe cardiovascular involvement, severe orthostatic hypotension, with involvement of the genitourinary, digestive and sudomotor systems.

Because they do not have central neurodegenerative symptoms, brain imaging tests in pure autonomic failure are normal and plasma catecholamine levels are normal or low, but do not show an adequate increase (>50%) with orthostasis, due to diffuse peripheral sympathetic denervation.

Some toxins can be causal factors, such as lead, thallium or arsenic poisoning, or use of some drugs such as chemotherapy drugs of the cisplatin class or vinca alkaloids, antiarrhythmic drugs such as amiodarone or vitamin deficiencies such as vitamin B12 deficiency.

Rare cases of family origin may occur, such as Hereditary Sensory and Autonomic Neuropathy (HSAN). These are divided into: Type I HSAN, which is lighter and starts in adult life, with distal sensory and autonomic involvement, and foot ulcers; type II HSAN, rarer, starting in childhood, with more diffuse and severe impairment.^8^^,^^19^^,^^31^^,^^33^

Autoimmune etiologies can justify various acute and subacute clinical presentations of pandysautonomia, with some similarities with Guillain-Barré syndrome (GBS). However, in acute pandysautonomia, somatic fibers are generally spared, unlike GBS. Some degree of autonomic dysfunction is also present in most cases of GBS.^31^^,^^34^^,^^35^

### Amyloidosis

Amyloidosis may occur in the following forms:

1. In the most common form, known as light chain (AL) or primary amyloidosis, there is abnormal clonal proliferation of plasma cells. Initially, peripheral sensitive distal neuropathy progresses to broad fibers, with subsequent autonomic failure of multiple affected organs, such as the digestive system, including esophagus and intestine, sudomotor system with alternating anhidrosis with compensatory sweating, renal involvement and nephrotic syndrome and cardiac involvement, with heart failure, arrhythmia and sudden death. In the autonomic evaluation, impairment of the sympathetic and parasympathetic systems can be found.
2. Familial amyloidosis (FA), also called paramyloidosis or Corino Andrade's disease,^36^^,^^37^ is found in the autosomal dominant form, originally described by Portuguese professor Dr. Corino de Andrade, in 1952. It has a higher incidence between 20 and 40 years of age, evolving to death at 10–12 years.
  It has a variable phenotype, depending on the geographic region and the mutation. Several forms have been described, such as: Portuguese (type I) or Andrade, Rukovina or Indiana (type II), van Alien (type III) and the Finnish type (type IV). In Brazil, some forms of this pathology have been described.^38^
  Mutation in the transthyretin (TTR) gene is the best known and studied, with various mutations described in this gen. It begins with symptoms of peripheral neuropathy, which can progress to severe generalized autonomic dysfunction, in addition to cardiological, neurological (sensorimotor peripheral polyneuropathy), visual, genitourinary, renal and gastrointestinal symptoms. Early detection is extremely important, aiming at treatment and preventing progression. Liver transplantation before the disease is advanced can change its course. New promising drugs have been launched, such as Tafamidis (TTR stabilizers), available in Brazil, and Inotersen.
3. The secondary form (AA form) is due to chronic pathologies, such as rheumatoid arthritis, osteomyelitis, tuberculosis, renal failure and its evolution depends on the control of the underlying disease.

Cardiac amyloidosis is mainly caused by AL or transthyretin-type FA (ATTR) or by deposition of wild-type transthyretin protein, once called senile cardiac amyloidosis. TTR deposits were found in 16% of patients with degenerative aortic stenosis and in up to 17% of patients with preserved ejection fraction heart failure. The prognosis after cardiac involvement is poor, with survival ranging from 2.5 to 3.6 years. On significantly increased left ventricular wall thickness (>14 mm), despite its low voltage, electrocardiography may suggest the diagnosis, complemented by cardiac nuclear magnetic resonance imaging and technetium pyrophosphate scintigraphy.^39^

The randomized study ATTR-ACT, evaluating the safety and efficacy of Tafamidis in patients with cardiac amyloidosis, revealed a reduction in all causes of mortality and hospital admissions after 30 months of follow-up, so Tafamidis started to be prescribed in this pathology, for NYHA (New York Heart Association) functional class (FC) I, II and III heart failure, mainly in the early stages. This was the first therapy to show improved survival of these patients.^40^

In many cases of dysautonomia, reports of recent viral infections are identified, especially by herpesviruses, Epstein-Barr and Coxsackie. Autoantibodies to ganglionic acetylcholine receptors (AChr) were found in 50% of patients with PAF, in 7% of patients with POTS and 0% in controls. The absence of these antibodies does not rule out the diagnosis. Case reports have demonstrated therapeutic success with the application of immunoglobulins in some of these clinical situations.^31^^,^^34^^,^^35^^,^^41^^-43^

In paraneoplastic syndromes, more commonly in small-cell lung carcinomas, the presence of autoantibodies, especially anti-Hu or ANNA-1, is usually present and clinical presentations are usually acute or subacute.

The autoimmune theory is reinforced by the appearance of symptoms after viral conditions, feverish conditions, after vaccination and in patients with previous autoimmune diseases, such as Hashimoto's thyroiditis, celiac disease and systemic lupus erythematosus.

Studies have shown that the autoimmune theory may be the pathophysiological mechanism of the “idiopathic” forms of some dysautonomic syndromes, such as pure autonomic failure (PAF), POTS or chronic fatigue syndrome.^43^

Anti-nicotinic cholinergic receptor antibodies have also been described. Authors have recently demonstrated the mechanism by which autoantibodies cause vasodilation and tachycardia. These findings may have important therapeutic implications. In the presence of anti-acetylcholine antibodies, the use of drugs such as pyridostigmine may be beneficial. In the presence of adrenergic antibodies, beta-blockers could be the best choice.

### Chagas Disease

Cardiac dysautonomia is well established in Chagas disease (ChD), in which anatomical denervation and functional abnormalities have been described in in vivo, post-mortem and experimental studies.^44^^-46^ Carlos Chagas’ original studies already called attention to the absence of a chronotropic response to atropine in patients with Chagas disease.^47^ In addition to denervation, other autonomic nervous system abnormalities, such as ganglionitis, neuritis, fibrosis, atrophy and fragmentation of specialized fibers have also been reported.^48^

Parasympathetic impairment can be detected in all forms of ChD, including the indeterminate and independent phase of left ventricular function.^49^^,^^50^ These data were corroborated by a meta-analysis that included seven studies that evaluated cardiac autonomic modulation, using R-R variability during the Valsalva maneuver.^51^

Studies with metaiodobenzylguanidine I-^123^ (^123^I-MIBG) have detected indeterminate form sympathetic dysfunction in Chagas disease patients without left ventricular systolic dysfunction.^52^^,^^53^ ^123^I-MIBG scintigraphy was also used to assess the presence and magnitude of sympathetic dysfunction in patients with Chagas cardiomyopathy and ventricular dysfunction (EF≤45%). The authors observed decreased ^123^I-MIBG uptake, indicating dysfunction of sympathetic receptors and loss of integrity of the presynaptic sympathetic fibers.^52^

An aspect that requires further clarification is the role of immune-mediated mechanisms in Chagas cardiomyopathy. In fact, many studies have demonstrated the presence of antibodies that react with cardiac muscarinic M2 receptors and B1 adrenergic receptors in the serum of asymptomatic Chagas disease patients.^48^^,^^54^

These autoantibodies could play a role in the pathogenesis of Chagas myocarditis, explaining cardiac neuromyopathy, described in the indeterminate phase.

Another topic that is poorly evaluated in Chagas dysautonomia is the investigation of orthostatic hypotension. In the ELSA-Brasil study, patients with positive ChD serology had a greater association with orthostatic hypotension (OR=2.29 — 95% CI: 1.2–4.2).^55^ In fact, there are inconsistent results in the evaluation of vascular control in Chagas disease patients (8). In contrast to other disorders with wide ANS involvement (for example, DM and amyloidosis), the presence of orthostatic hypotension in ChD is not usually described.^44^^,^^56^

Early autonomic impairment in ChD suggests that cardiovascular dysautonomia may be associated with increased morbidity and mortality, cardiac arrhythmia and sudden death.^49^^,^^52^ It could be one of the central pillars in several clinical manifestations, such as diastolic and/or systolic dysfunction, ventricular dilation, tachyarrhythmia and bradyarrhythmia and sudden cardiac death.^45^^,^^50^^,^^53^ Cardiac autonomic dysfunction must be a determinant or a predisposing pathophysiological risk factor in the genesis of arrhythmia. Greater arrhythmogenic vulnerability is observed in cases with more focal autonomic dysfunctions than in cases with more diffuse and significant injuries, due to a greater degree of central nervous system disconnection, with less susceptibility to ANS interference in cardiac electrophysiological properties.^45^^,^^57^

The observation of sustained ventricular tachycardia in patients with Chagas cardiomyopathy, with preserved ventricular function and regional myocardial sympathetic denervation (detected by ^123^I-MIBG scintigraphy), as well as during orthostatic stress in a patient with mild impairment of ventricular function and no significant baseline electrocardiographic abnormalities lead to an alleged role of autonomic dysfunction in the pathophysiology of rhythm disorders in Chagas cardiomyopathy.^53^

**Orthostatic Hypotension — A Sign of Late Stage and Severity**

The detection of neurogenic orthostatic hypotension (nOH) usually represents a late stage and severity, correlated with worse prognosis. Therefore, one should not wait for its presence for diagnosis of dysautonomia. Patients with known pathologies or symptoms that compromise the ANS should be investigated early.

## Classification of Clinical Syndromes

### Cardiovascular Autonomic Neuropathy (CAN)

CAN is a term widely used by the Societies of Diabetes and Autonomic Neuropathy to express impairment of the cardiovascular autonomic nervous system in the presence of diabetes mellitus, but the term is not restricted to this pathology.^7^ CAN includes ANS involvement, from the pre-clinical stage, which may have prognostic implications, such as glucose intolerance or pre-diabetes. (Figure 2)

**Figure 2:** Causes of dysautonomia.

Source: prepared by the author. The image includes examples as different causes of acute or chronic, central or peripheral autonomic dysfunctions. Some pathologies can present in different ways. POTS: Postural orthostatic tachycardia syndrome.

The expression neurogenic orthostatic hypotension, widely used by arrhythmologists and cardiologists, links the need for the presence of OH to define the diagnosis, a situation that, when detected, may represent a late and more severe stage, often with irreversibility of the condition.

### Neurogenic Orthostatic Hypotension (nOH) and Supine Hypertension

Orthostatic hypotension is defined by the presence of reduced systolic blood pressure (BP) of at least 20 mmHg or diastolic BP of 10 mmHg or both, within 3 minutes after active orthostatic position or during the tilt test.^3^

In patients with nOH, impairment of the autonomic nervous system is observed, characterized by the inability to provide adequate vasoconstriction and/or adequate compensatory increase in heart rate (HR), sufficient to maintain BP in an orthostatic position. In most cases, this dysfunction is attributed to the insufficient release of norepinephrine from the sympathetic nerves.^42^^,^^43^

While in nOH impaired vasoconstriction is due to permanent damage in the efferent sympathetic activity, in non-neurogenic orthostatic hypotension (nnOH), it includes a variety of causes, such as the use of medications, antihypertensives, antidepressants, and alpha-blocking agents (Table 1), in addition to volume depletion and chronic diseases that lead to physical deconditioning.^58^

**Table 1:** Medications that can cause orthostatic hypotension or exacerbate symptoms of neurogenic orthostatic hypotension

Class of medications	Examples
Dopaminergic drugs	Levodopa, dopamine agonists
Tricyclic antidepressants	Amitriptyline, nortriptyline
Anticholinergics	Atropine
↓ Pre-load Diuretics Nitrates Phosphodiesterase inhibitors	Furosemide, hydrochlorothiazide, spironolactone Isosorbide dinitrate Sildenafil, vardenafil
Vasodilators Alpha-1 adrenergic antagonists Ca++ blockers Direct vasodilators	Doxazosin, tamsulosin Amlodipine, nifedipine Hydralazine
Negative inotropes and chronotropics Beta-blockers Non-dihydropyridine calcium channel blockers	Propranolol, metoprolol, atenolol, bisoprolol, nebivolol, carvedilol Diltiazem, verapamil
Central action sympatholytics	Clonidine, methyldopa
Renin-angiotensin system antagonists Converting enzyme inhibitor Angiotensin blockers	Captopril, enalapril, perindopril Losartan, telmisartan, candesartan

Source: Adapted^4^

It is important to differentiate nOH from nnOH due to the worse prognosis of nOH, with greater morbidity and mortality from all causes. Furthermore, studies point out that the presence of OH in middle-aged individuals predisposes to myocardial hypertrophy even in the absence of hypertension.^58^^,^^59^ The incidence of OH increases with age, as well as hypertension, diabetes and cardiovascular or degenerative diseases.^42^^,^^43^^,^^59^

Patients with one of the five categories below are at increased risk for nOH compared to the general population and should be routinely investigated (Figure 3):

1. Suspected or diagnosed with any degenerative disease associated with autonomic dysfunction, including Parkinson's disease, multiple system atrophy, pure autonomic failure or dementia by Lewy bodies;
2. History of unexplained falls or syncope;
3. Presence of peripheral neuropathy;
4. Age ≥70 with a high degree of fragility or use of multiple medications;
5. Dizziness or unspecific orthostatic symptoms.

**Figure 3:** Flowchart of Evaluation and Follow-up of Dysautonomia or Cardiovascular Autonomic Neuropathy (CAN) It occurs idiopathically, as in multiple system atrophy or pure autonomic failure, or in pathologies such as diabetes mellitus, neurodegenerative diseases, Parkinson's disease, dementia syndromes, chronic renal failure, amyloidosis, some neoplastic diseases and in the elderly.Electrocardiogram to assess heart rate and QT interval.Neurological tests such as electromyography, brain resonance imaging, cardiological tests such as 24-h Holter, ischemia evaluation.Laboratory tests including complete blood count, renal function, cortisol, ACTH, glycemic profile, plasma catecholamines collected lying down and immediately after orthostasis, neoplastic and autoimmune disease markers, and others (see specific section).As recommended by international guidelines on diabetics.^1^^,^^2^^,^^7^SGLT2 — Diabetes medications — sodium-glucose co-transporter inhibitors used to treat diabetesPatients with very frequent extrasystoles, atrial fibrillation, cardiac pacemaker and advanced cognitive dysfunction cannot be evaluated using this methodology. Consider that several drugs must be suspended for the examination and the values of the measurements must be correlated with normal values for age and sex.

Source: prepared by the author.

After identifying that a patient is at risk for orthostatic hypotension, it is important to measure BP and HR in the supine position (after 5 minutes lying down) and in the first and third minutes after the orthostatic position, which is considered the gold standard for OH diagnosis.^58^ These values must also be measured after 5 minutes of orthostasis.

An alternative method would be taking these measurements after the patient has been 5 minutes in the sitting position, then after 3 minutes in the orthostatic position. Many of these patients still have supine hypertension (systolic BP ≥140 mmHg and/or diastolic BP≥90 mm Hg). In this situation, it is recommended to consider OH if there is a drop in systolic BP≥30 mmHg and/or diastolic BP≥10 mmHg.^58^

HR measurements also vary from supine position (and/or sitting position) to orthostatic position and help to differentiate nOH from nnOH.^42^^,^^59^ In individuals with OH, a compensatory HR increase of at least 15 bpm is expected within 3 minutes in the standing position. If this does not occur, OH is possibly neurogenic (as long as there is no concomitant use of negative chronotropic medication or conduction system disease or patient with a pacemaker).

A review of the prescribed drugs should be carried out in order to avoid effects on the baroreflex response (table 1), especially alpha- and beta-adrenergic blockers and centrally acting alpha-2 agonists.

Some patients may have postprandial hypotension, particularly after large meals rich in carbohydrates, combined with alcoholic beverages. In these conditions, BP measurements in the supine and orthostatic position should be performed before and after the meal, which can usually occur up to 90 minutes after the meal.

Symptoms of orthostatic intolerance may occur in patients without orthostatic hypotension detectable on clinical examination due to impaired peripheral vasoreactivity and venous return. In these cases, reduced stroke volume is observed during hemodynamic monitoring in the orthostatic tilt test. The compensatory HR response is sufficient to maintain blood pressure at acceptable levels.^59^^,^^60^

Complementary investigation (table 2) is applied to uncover potential non-neurogenic causes of OH.^58^

**Table 2:** Investigation of patients with orthostatic hypotension (OH)

Diagnostic tests	
Electrocardiography	Evaluate rhythm and conduction disorders, hypertrophy, low voltage
Complete blood count	Evaluate anemia and/or infection
Metabolic profile (sodium, potassium, calcium, creatinine, urea, fasting glucose, glycated hemoglobin, bicarbonate); Urinary sodium in 24 hours	Volume depletion (urea/creatinine ratio >20 mg/dl; kidney failure or diabetes or metabolic disorders
TSH, free T4, Cortisol, ACTH, vitamin B12	Thyroid and adrenal dysfunction and vitamin B12 deficiency
Serum albumin	Malnutrition and chronic disease
Enzymes and liver function	In patients with weight loss, suspected alcoholism
Study of autoantibodies (ANNA-1; ANNA-2, Anti-AChr, LGI1, and others) in cerebrospinal fluid and/or blood	Recent onset OH, suspected paraneoplastic syndrome, pure autoimmune autonomic failure
Serum and urinary protein electrophoresis, protein immunofixation Nerve biopsy, abdominal fat with Congo red stain	In patients with peripheral neuropathy, suspected amyloidosis
Plasma catecholamines in decubitus and after orthostasis	Pure autonomic failure
Serology for arboviruses (dengue fever), for COVID-19, HIV serology	Investigation according to clinical history
Investigation for collagenoses (autoantibodies such as FAN, anti-DNA, anti-SM, Anti-RNP)	Suspected collagenoses

Source: Adapted;^58^ Anti-AChR: Autoantibodies to ganglionic acetylcholine receptors (AChR); ANNA: anti-neuronal nuclear antibodies; anti-RNP: anti-ribonucleoprotein antibodies; HIV: acquired immunodeficiency virus. COVID-19: Infection with the new coronavirus has been associated with dysautonomic forms such as the chronic fatigue syndrome.

If the standardized blood pressure measurements for the diagnosis of OH are not effective for the diagnosis, other approaches can be taken:

1. Advise the patient to measure BP and HR at home in different situations:
  - Fifteen minutes after going to bed at night or before getting up in the morning;
  - Three minutes after taking an orthostatic position, before taking medication or whenever symptoms appear;
2. Perform the orthostatic tilt test, which can document an early or late OH;
3. Perform 24-hour ambulatory blood pressure monitoring (ABPM) — the patient should take notes on lying down and getting up.

When diagnosis of OH is confirmed, it is important to establish the severity, which depends on the magnitude of the drop in systolic BP, the time of tolerance in the orthostatic position and the magnitude of the symptoms to daily activities.

A grading scale from 1 to 4 (table 3) was proposed as a stratification of these patients. For grades 3 and 4, it is advisable to refer the patient to a center specializing in the treatment of orthostatic hypotension.^61^

**Table 3:** Grading scale for the severity of neurogenic orthostatic hypotension

Grade	Signs and symptoms
1	Infrequent symptoms/no restriction to stand upright, with 20 to 30 mm Hg drop in SBP
2	>30 mmHg drop in SBP upon orthostasis time ≥5 min
3	>30 mmHg drop in SBP upon orthostasis time <5 min or severe impact on daily activities
4	>30 mm Hg drop in SBP in <1 min in orthostasis or functional incapacity.

Source: Adapted.^61^

Orthostatic hypotension may be present in only 30–50% of patients with pure autonomic failure and in 60–70% with multiple system atrophy.^33^

## Pandysautonomia and Evaluation Scores

Many pathologies can promote the global involvement of the ANS, with impairment of various systems and organs.

It is called pandysautonomia when there is evidence of systemic dysautonomia: cardiovascular dysautonomia and dysautonomia of various organs. Patients with cardiovascular autonomic neuropathy and/or neurogenic orthostatic hypotension should be asked about specific symptoms in other systems.

Some questionnaires can be used for better clinical evaluation, such as the ASP (Autonomic Symptom Profile), which contains 73 questions and the COMPASS (Composite Autonomic Symptom Scale), which uses the previous scale and quantifies the severity of abnormalities. Validation of these questionnaires has not been done in different clinical contexts. However, the items that comprise it can be used as a screening tool in the suspicion of impairment of other organs. ^61^^.^^62^

More recently, a new Survey of Autonomic Symptoms (SAS) score was developed and validated, showing better sensitivity in detecting mild autonomic neuropathies, not requiring complementary methods, and it can be a good clinical tool for early detection of autonomic neuropathy (Table 4).^61^

**Table 4:** Survey of Autonomic Symptoms (SAS) questionnaire to diagnose the involvement of different organs and systems in dysautonomia

Symptoms/Health Problem	Have you had any of these symptoms in the last 6 months? 1- Yes; 2- No	How severe is this symptom? Scale of 1 to 5 (used if symptoms are present)
1-Darkened vision?	1 or 2	1 – 5
2-Dry mouth or dry eyes?		
3-Pallor or cyanosis?		
4-Feeling cold in some regions of the body?		
5-Reduced feet sweating compared to the rest of the body?		
6-Reduced or absent feet sweating after exercising or in hot weather?		
7-Increased hand sweating compared to the rest of the body?		
8-Nausea, vomiting or gas after light meals?		
9-Diarrhea (>3 bowel movements per day)?		
10-Persistent constipation?		
11-Loss of urine?		
12-Erection issues?		

Source: Adapted.^61^ The presence of 3 or more symptoms resulted in 95% sensitivity and 65% specificity, while the presence of 7 or more points determined 60% sensitivity and 90% specificity. Gastrointestinal symptoms were less correlated with other indexes.

## Chronic Fatigue Syndrome

It is currently considered a chronic systemic disease that profoundly affects the quality of life of patients. It has been called chronic fatigue or myalgic encephalomyelitis due to the documentation of central and autonomic nervous system abnormalities. This syndrome affects about 2.5 million individuals of all ages in the USA and dramatically reduces productive capacity.

It is a complex disease that involves deregulation of the central nervous system, the immune system, with dysfunction of the cellular energy metabolism and ionic transport, in addition to cardiovascular abnormalities. It is characterized by persistent and recurrent fatigue after exercise, with no other cause that explains the origin of the symptoms (table 5).^9^^,^^63^^–^^66^

**Table 5:** Classical Criteria for the Diagnosis of Chronic Fatigue Syndrome

Extreme, persistent or recurrent tiredness, without a justified cause, with the following characteristics:	
1. Recent onset (that is, non-progressive throughout life) or with specific trigger	
2. Difficulty performing usual professional, physical or social activities	
3. Meeting at least 4 of the following criteria:	
	3.1. Impaired concentration and recent memory
	3.2. Sore throat
	3.3. Cervical or axillary lymph nodes
	3.4. Joint and muscle pain
	3.5. Headache
	3.6. Non-restorative sleep
	3.7. Post-exertional malaise persisting for >24 hours

Source: Adapted^9^

Routine laboratory tests are usually normal. Impaired autonomic regulation of the vascular system is commonly found, especially in deficient response to orthostatic position, resulting in high association with dysautonomia (figures 4 and 5).

**Figure 4:** Tilt test with hemodynamic measurements, where systolic volume, cardiac output and peripheral vascular resistance (PVR) were corrected for body surface, resulting in systolic index (SI), cardiac index (CI) and PVR index (PVRI). 4A. Patient with clinical diagnosis of chronic fatigue. Right after tilting, there is an exaggerated reduction in SI (>30%), initially compensated by the expected increase in PVRI and HR. After 15 minutes of tilting, there is a greater SI reduction associated with a PVRI reduction, instead of the greater compensatory increase expected of the PVRI. Therefore, the compensation to keep the BP stable occurs at the expense of a greater HR increase, which then presents excessive increase (>30 bpm), than in the supine position. This change occurs later (10 minutes after the beginning of the test), not fulfilling the criteria for POTS. 4B. Patient diagnosed with POTS. During the tilt test, an SI reduction is not compensated by a PVRI increase. PVRI decreases, rather than increases, in orthostatic position. Therefore, mean blood pressure (BP) remains stable due to an excessive increase in heart rate (HR) by >30 bpm, occurring in the first 10 minutes of tilting, associated with symptoms, thus fulfilling the POTS criteria. The difference between the two conditions can be, in some cases, only time-related.

**Figure 5:** Tilt test with hemodynamic measures, where systolic volume, cardiac output and peripheral vascular resistance (PVR) were corrected for body surface, resulting in systolic index (SI), cardiac index (CI) and PVR index (PVRI). BP — blood pressure 5A. Patient with orthostatic intolerance. There is no expected PVRI increase. Instead, it presents a progressive reduction compensated by a progressive increase in HR, until the end of tilting, with a slight reduction in BP. The symptoms occur in the presence of a deficit in PVR increase in an orthostatic position. 5B. Patient with late orthostatic hypotension. In this case, there no SI reduction and there is a progressive PVRI reduction during the tilt test. After 10 minutes of tilting, when there is a greater PVRI reduction, which is not accompanied by any additional HR increase, orthostatic hypotension is observed, with symptoms. After 20 minutes, spontaneous recovery of PVRI and BP occurs, with relief of symptoms.

Neuroinflammation can have different triggering factors: brain infection (chronic herpes virus), autoantibodies, neurotoxins or chronic stress, and extra-cerebral inflammatory processes, including the intestine. Low levels of neuroinflammation trigger protective behavioral abnormalities, such as reduced activity, reduced appetite and increased sleep.^63^^–^^66^

Functional magnetic resonance imaging in patients with chronic fatigue demonstrated different responses to visual and auditory stimuli and memory tests, as well as abnormalities in connectivity between areas of the brain. Positron emission tomography demonstrated widespread neuroinflammation and high lactate levels, which correlate with degrees of fatigue. In the spinal fluid, there is a higher rate of proteins related to injury and muscle repair.^65^^,^^66^

Metabolic abnormalities have also been described, resulting in impaired generation of cellular energy from different sources: oxygen, sugar, lipids and amino acids, with high levels of oxidative stress and nitric acid. Many metabolites are found to be below normal levels. This hypometabolic condition is observed in some animals in hibernation and allows animals under threat to slow down the metabolic process of energy consumption to preserve vital functions.^65^^,^^66^

Abnormalities of the autonomic nervous system include abnormal heart rate and blood pressure during prolonged orthostatic position, which are not sufficient to deliver diagnosis of POTS, or orthostatic hypotension, but are associated with reduced cerebral flow and cause symptoms.

In provocative tests of physical, orthostatic and mental challenges, various symptoms are observed, especially after 12 to 24 hours of activity, known as “post-exertional malaise.” Patients still have difficulty extracting oxygen during exertion, resulting in reduced anaerobic threshold.^67^

In the last decade, there has been an alarming increase in patients with other associated morbidities, such as chronic pain and functional impairment.^46^^–^^47^ The same diagnostic criteria can be applied: chronic fatigue, chronic pain including headache, sleep disorders, mood disorders, post-exertional malaise, orthostatic and exercise intolerance and difficulty maintaining the usual functional capacity before the onset of symptoms.

Orthostatic intolerance is defined by the presence of dizziness, light head, visual turbidity and pre-syncope, which get worse in orthostatic position and are alleviated with horizontal posture.

Chronic diseases associated with chronic fatigue, as well as chronic fatigue alone, typically occur after a triggering event: Viral, bacterial or fungal infection, surgery, car accident, pregnancy, vaccination or after a prolonged period of physical or mental stress. Recently, infection with the new coronavirus (COVID-19) has been shown to affect several areas of the nervous system, with suspected cases of chronic fatigue being reported, causing concern about the possibility of a marked increase of this condition.^68^^–^^72^

In some cases, no precipitating factor is identified, but there may be family history of similar symptoms in first-degree relatives, suggesting a genetic component. Many patients develop anxiety and depression secondary to chronic diseases or as part of the pathophysiological abnormalities of the underlying disease. A significant number of patients have autoimmune and inflammatory markers.

Objective findings include: orthostatic intolerance to the tilt test, autonomic dysfunction and small fiber neuropathy (in autonomic function tests), hypovolemia and abnormality on functional magnetic resonance imaging (MRI) tests, single photon emission computed tomography (SPECT) or positron emission tomography (PET scan). Conventional MRI shows only non-specific findings.^69^^–^^71^

Despite recent discoveries, there is no highly sensitive and specific method for an accurate diagnosis yet, as there is no effective treatment.

As part of the treatment of chronic diseases associated with chronic fatigue, psychotherapy, cognitive behavioral therapy, and occupational therapy can improve the functional state and reduce the suffering of these patients. Medications are generally used for headache, neuropathic pain, muscle tension, gastrointestinal symptoms and sleep disorders. It is extremely useful to separate the different etiologies of chronic fatigue.

Mast cell activation syndrome can cause symptoms of chronic fatigue or POTS. In that case, antihistamines can be useful. In connective tissue diseases, anti-inflammatory drugs, immunomodulatory therapy such as chloroquine or intravenous immunoglobulin and corticosteroids can be used to control joint pain and fatigue.

**Chronic Fatigue Syndrome — New criteria^8^**

It has been recently recommended that chronic fatigue be renamed Systemic Exertion Intolerance Disease, with new diagnostic criteria:

1. Unexplained fatigue and consequent occupational disability for more than 6 months;
2. Post-exertional malaise;
3. Non-restorative sleep;
4. Cognitive impairment or orthostatic intolerance.

## Postural Orthostatic Tachycardia Syndrome (POTS)

It is defined as an exaggerated chronotropic response to the change from horizontal posture to orthostasis, persistent and associated with symptoms of orthostatic intolerance (OI).^73^^,^^74^ It is the most common cause of OI in the young population. It affects about 500,000 to 3,000,000 individuals in the United States alone, the majority of whom are females (4:1), aged 15 to 25 or at the beginning of their professional lives.^10^^,^^11^^,^^75^ Sustained heart rate (HR) increase ≥30 bpm (≥40 bpm if <20 years old) or HR ≥120 bpm is observed in the first 10 minutes in an orthostatic position or during the tilt test, with no classical orthostatic hypotension associated. A slight decrease in blood pressure may occur.

Generally, one or more triggering factors are identified: acute stress such as pregnancy, surgery, previous infection, vaccine or traumatic event. Among the most common infections are: the mononucleosis virus (18.6%), respiratory (18%) and gastrointestinal (11.4%) viruses.^10^^,^^76^^,^^77^

In a preliminary evaluation of patients with suspected POTS, in addition to history taking and physical examination, vital signs must be taken in a supine and orthostatic position. Clinical history aims to investigate the potential causes of orthostatic tachycardia, including potential triggers. POTS symptoms are usually exacerbated by exercise, hot weather, dehydration and alcohol intake.

Electrocardiography and ambulatory ECG monitoring should be performed to rule out potential primary causes of tachycardia and echocardiography and exercise test to check for structural heart disease and heart rate response to exertion. Thyroid function tests, as well as blood count, should be part of the investigation routine, to rule out secondary causes of tachycardia.

The orthostatic tilt test can be useful to obtain hemodynamic parameters and tolerance to orthostatic position. Extended autonomic evaluation, with analysis of various hemodynamic parameters during the tilt test, is highly recommended in the investigation and differential etiological diagnosis of POTS.

Continuous and non-invasive BP and ECG monitoring systems, associated with bioimpedance measurements, allow to evaluate systolic volume, peripheral vascular resistance and cardiac output, making it possible to identify the type of hemodynamic disorder found in patients with POTS (Figures 4 and 5).

POTS is a heterogeneous syndrome resulting from different non-excluding pathophysiological mechanisms. It can be classified into five types, according to the prevailing pathophysiological mechanism: Neuropathic, hypovolemic, hyperadrenergic, secondary to noradrenaline abnormalities or activation of mast cells, and related to joint hypermobility (Ehlers-Danlos syndrome).^76^^–^^81^

In the neuropathic form, the main mechanism is impairment of peripheral vasoreactivity due to predominantly sympathetic denervation. In these cases, blood volume accumulates in the lower limbs in an orthostatic position and sympathetic system activation results in reflex tachycardia, which is not always compensatory. About 50% of these patients also have peripheral sudomotor denervation, suggesting post-ganglionic sympathetic denervation.

In the hypovolemic form, 70% of patients have hypovolemia due to excessive fluid retention in the lower compartment of the body. There is reduced tone, increased venous capacitance and reduced systolic volume during the tilt test. This central hypovolemia results in adrenergic activation by the baroreceptors and exacerbated compensatory reflex tachycardia.

Many patients in this group have reduced total blood volume, both in plasma and in blood cells.^78^^,^^79^ Paradoxically, some of these patients have low levels of plasma renin and aldosterone activity and high levels of angiotensin II.^78^

In the Hyperadrenergic form, excessive adrenergic activation causes symptoms that include palpitations, sweating, tremors, anxiety and even hypertension triggered by physical activity or emotional stimulation. The primary hyperadrenergic form is characterized by high levels of plasma norepinephrine due to higher production (1000–2000 pg/ml), occurring in 5 to 10% of the cases.

The secondary form consists of a heterogeneous group divided into 3 main categories:

1. reduced clearance of synaptic norepinephrine (mutation of loss of function);
2. mast cell activation disorder — characterized by the presence of high urinary methylhistamine;
3. pharmacological blockade of norepinephrine transport by drugs that inhibit this transport, such as tricyclic antidepressants and other amphetamine-like drugs, the latter being the most frequently found type.

In the Ehlers-Danlos Syndrome, a connective tissue disease, with skin hyperelasticity and joint hypermotility, 70% of individuals have POTS and 18% of patients with POTS have diagnostic criteria for the Ehlers-Danlos syndrome, considered an underlying mechanism for the syndrome.^80^

In cases of patients with POTS with the mast cell activation syndrome, an autoimmune factor may be present. These patients have flushed skin and hypertension associated with orthostatic tachycardia. It is not yet clear whether sympathetic activation causes mast cell degranulation or whether mast cell activation causes vasodilation.^80^^,^^82^

In refractory patients, an extensive evaluation at a center specializing in autonomic tests should be considered. Valsalva's maneuvers with beat-to-beat BP measurement may show an exaggerated phase 4, revealing excessive sympathetic activity. Measurement of plasma epinephrine and norepinephrine in a supine and orthostatic position can be useful to identify hyperadrenergic cases, as well as analysis of 24-hour urinary sodium in cases of volume depletion.^6^

Anxiety and hypervigilance are often common in patients with POTS. However, HR increase is not due to an anxiety condition, but due to a physiological abnormality. Still, psychological assessment and follow-up can be useful in the clinical management of these patients.

Physical deconditioning is common to all forms of POTS. Multiple parameters associated with deconditioning are present in these patients: reduced cardiac area and mass (16%), reduced blood volume (20%) and reduced peak oxygen consumption (VO^2^), compared to sedentary controls. Both bed rest and deconditioning reduce the baroreflex sensitivity to produce vasoconstriction.

In a study for international registration of POTS, progressive physical conditioning showed volume expansion and increased the cardiac area of patients, resulting in a significant improvement in symptoms. In this study, 71% of patients who completed the training program were free of POTS diagnosis. In a small group followed up for 6 to 12 months, the result was also maintained.^83^

The protocol consisted of 8 months of progressive training with aerobic exercise (3 sessions per week) associated with 2 weekly sessions of low-resistance muscle strengthening exercise, starting in the supine position and progressing to the orthostatic position. Compared to beta-blockers, exercise showed improved quality of life and normalized neurohumoral response, being considered class IIa of indication in international guidelines.^11^^,^^83^^,^^84^

There is no class I recommendation for the treatment of POTS. Non-pharmacological measures include increasing fluid intake to 2–3 liters/day and salt to 10–12 grams/day. Infusion of up to 2 liters of saline is recommended for acute decompensations (class IIb).^11^

If non-pharmacological measures are not effective, pharmacological treatment can be established according to the type of disorder identified (fFgures 4 and 5) or the modified algorithm proposed by Bryarly et al. (Figure 6).^74^

**Figure 6:** Treatment algorithm for patients with POTS.

Source: adapted.^74^

**Chronic Fatigue Syndrome x Postural Orthostatic Tachycardia Syndrome (POTS)**

Postural orthostatic tachycardia syndrome (SPOT) has been found in 29% of patients with chronic fatigue syndrome, while almost 50% of POTS patients have chronic fatigue syndrome.

Fludrocortisone may be useful in volume expansion, but its effect has not yet been tested in large clinical studies. Midodrine is an alpha-1 adrenergic agonist that increases the contraction of veins and arteries. This medication significantly reduces HR, but to a smaller extent than saline infusion. It has fast action and metabolization time and should be used 3 times a day, while the patient is active, avoiding potential nighttime hypertension.

Medications such as midodrine associated with a low dose of non-selective beta-blocker (propranolol), fludrocortisone and pyridostigmine are useful in the dysautonomic and hypovolemic forms of POTS. In the hyperadrenergic form, clonidine or alpha-methyldopa can be effective (class IIb).^11^

Sinus node modification by radiofrequency is not recommended and may be harmful, as it eliminates the compensatory mechanism of low cerebral output, which is sinus tachycardia, triggered by the baroreflex action.

Concomitant symptoms, such as headache and sleep disorders or gastrointestinal problems are often seen in POTS, and should be treated appropriately, as well as cognitive behavioral therapy should be considered.

## Carotid Sinus Hypersensitivity and Cardioneuroablation

The prevalence of carotid sinus hypersensitivity (CSH) varies with age. It is extremely uncommon in individuals aged <50 and exponentially increases with age. In patients with syncope and age over 60, an abnormal carotid sinus response has been observed in up to 22.3%. Therefore, it is a common finding in elderly patients without syncope, especially if they have cardiovascular disease. For this reason, there is a consensus that for the diagnosis of carotid sinus hypersensitivity syndrome there is reproduction of clinical symptoms during carotid sinus massage and previous history of spontaneous syncope, suggestive of reflex origin.^12^^,^^85^^,^^86^ Positive carotid sinus massage, but no history of syncope, only defines carotid sinus hypersensitivity and not the clinical syndrome (Table 6).

**Table 6:** Definition of Carotid Sinus Hypersensitivity

DEFINITION	
Reduced heart rate and/or blood pressure (BP) in response to carotid sinus massage:	
	1. Cardioinhibitory: pause ≥3 seconds (usually >6 seconds);
	2. Vasodepressor: drop in BP ≥50 mmHg, with no significant bradycardia;
	3. Mixed: pause ≥3 seconds associated with SBP drop ≥50 mmHg.

Source: Adapted^1^

Carotid sinus massage is a class I indication in international guidelines for patients >40 years, with syncope of unknown origin, compatible with reflex mechanism (class I).^12^ Massage is, however, controversial, as asymptomatic patients may present hemodynamic abnormalities with symptoms during maneuver.^87^ However, if the syncope is of undetermined origin and the response to carotid sinus massage, in the cardioinhibitory form, reproduces the clinical symptom, there is a presumptive cause of syncope, as the use of a pacemaker in this group of patients improved the symptoms of syncope in some studies.^11^^,^^88^

Perhaps the best way to confirm the cause of syncope in this context would be by long-term ECG monitoring (external or implantable looper). Although this technique (external or implantable looper) is more accurate to diagnose cases of carotid sinus hypersensitivity in the cardioinhibitory form, it would not be able to identify the vasodepressor forms of hypersensitivity.^89^

Carotid sinus massage should be preferably performed with continuous beat-to-beat BP and electrocardiogram monitoring. It is safer when performed at the tilt test facility. Maneuver should be performed with the patient's face rotated laterally, in a supine position and, if negative, it must be repeated in an orthostatic position, on each side, for a maximum of 10 seconds of compression, at the site of greatest carotid pulsation, at an angle formed by the mandible, the cricoid cartilage and the anterior margin of the sternocleidomastoid muscle. It should be avoided in patients with carotid murmur before adequate evaluation.

Although serious complications are rare (0.24%), the risk of transient ischemic attack must be considered, especially for patients who have previously experienced this event, as well as stroke or carotid artery stenosis >70%, as these are contraindications for the maneuver.^12^

The carotid sinus is a baroreceptor that responds to wall stretching, as with high BP.^65^ In this situation, there is increased vagal tone and reduced sympathetic tone. Otherwise, on reduced BP and reduced vascular wall tension, there is a reduction in baroreceptor triggers, resulting in attenuation of vagal action. Baroreflex stimuli are sent from the carotid sinus to the solitary tract nucleus, where a large number of cardiovascular neurons are located.

Although the physiology of the carotid sinus baroreflex is reasonably well understood, the pathophysiology of CSH remains unclear.

Three main pathophysiological mechanisms have been considered:^90^^–^^94^

Atherosclerosis: theoretically, reduced vessel compliance could result in reduced afferent flow of the baroreflex impulse. However, it has been shown that the afferent portion of the carotid sinus reflex is intact in individuals with CSH.

Sternocleidomastoid muscle denervation:^92^ with age, sternocleidomastoid muscle denervation (demonstrated by electromyography), thus reducing the information sent to the solitary tract nucleus, while the carotid sinus baroreceptors continue to send proper signals to the same nucleus, generating information imbalance. Thus, the head movement may result in afferent signals only from the carotid sinus, being interpreted by the solitary tract nucleus as an increase in BP, triggering an abrupt reduction in BP and HR.

Generalized autonomic dysfunction: high sympathetic activity has been recently demonstrated in individuals with CSH, symptomatic or asymptomatic, which suggests a generalized autonomic dysfunction.

The most common clinical manifestations of CSH are syncope, pre-syncope or dizziness during maneuvers with a change in head position. Loss of consciousness, as well as recovery, generally occur suddenly. Injuries resulting from falls are hence commonly observed.

Elderly patients may refer to episodes as recurrent falls, with no apparent cause. They may not report changes in head position during the fall.

Regarding treatment of the vasodepressor form of CSH, studies with midodrine^95^ and fludrocortisone^96^ showed improvement of syncope and presyncope symptoms compared to placebo. However, for patients with the cardioinhibitory form, definitive pacemaker implant has been the treatment of choice.

The decision to implant a pacemaker after a single episode of syncope will depend on the consequence and severity of the injury resulting from this episode. Some small observational randomized studies have shown improvement in clinical symptoms after implantation.^11^^,^^12^^,^^15^

However, randomized blinded studies comparing dual-chamber pacemakers versus dual-chamber pacemakers without active stimulation (off) did not show significant improvement in patients with unexplained falls.^88^^,^^90^^,^^97^^,^^98^ Neither do large-scale randomized studies testing the use of pacemaker in cardioinhibitory form, raising questions about the recommendations of the current guidelines.^97^ On the other hand, a meta-analysis of three studies showed 9% recurrence of syncope in patients with active stimulation, versus 38% in the control group (without a pacemaker).^99^ This meta-analysis and other review studies are the basis of support for current recommendations for pacemaker implantation with Class IIa indication level, in American^15^ and European^11^^,^^15^ syncope guidelines.

Carotid sinus denervation by irradiation or endarterectomy has also been considered in the past as a treatment option.^100^

Regarding the prognosis, there has been no difference in mortality between patients with and without CSH compared to individuals of the same age.^87^^,^^101^ However, the consequences of an injury resulting from a fall in an elderly patient cannot be adequately estimated. Therefore, patients must be informed that the risk of recurrent syncope should be reduced, but minor symptoms including pre-syncope may persist, even with therapies implemented.

Another very promising treatment strategy for reflex syncope resulting from exacerbated vagal activity is a technique known as cardioneuroablation, which consists of modifying vagal activity by catheter ablation, using radiofrequency energy.^102^

Pachon et al.^103^ observed that when nerve fibers mix with myocardial cells, they produce changes in their conduction, from compact (uniform conduction with main frequency of 40 Hz, which occurs around very well-connected cells) to fibrillar conduction (conduction with fractional potentials with a frequency greater than 100 Hz). The authors used the fibrillar myocardial pattern (found mainly in the region of the sinus node and atrioventricular node) as a marker of neuromyocardial interface and target sites for cardioneuroablation and achieved clinical improvement of syncope episodes.^103^

Exciting results have been described in the literature with fibrillar myocardial ablation around the sinus node and atrioventricular node. During the ablation procedure, the disappearance of high-frequency potentials in these areas resulted in improved sinus and nodal function.^104^

Cardioneuroablation has been used to treat patients with carotid sinus hypersensitivity and can be an alternative to implanting a pacemaker, especially in young individuals, as these are more vulnerable to long-term complications.^105^^,^^106^

In summary, ablation of ganglionic plexuses can promote a significant reduction in vagal activity, in the sinus and atrioventricular nodes, and is effective in reducing symptoms in patients with severe neuromediated bradycardia. Due to the different techniques employed, randomized multicenter studies would be necessary to define the effectiveness, the best technique, safety and reproducibility of the method.^107^

## Inappropriate Sinus Tachycardia (IST)

The first case of inappropriate sinus tachycardia (IST) was described in the literature in 1939 by Codvelle and Boucher.^108^ A prevalence of 1.2% is currently estimated in the general population.^11^ It is considered a chronic condition, but little is known about its evolution and mortality. Its mechanism is poorly understood,^109^^–^^113^ including increased sinus node automaticity, beta-adrenergic hypersensitivity, reduced parasympathetic activity and impaired neurohormonal modulation.

The onset of symptoms is usually associated with a stressful event, such as a divorce of parents of teenagers, separation or another major family event. The symptoms usually found are: palpitations, dizziness, and syncope. Abdominal discomfort, sweating, headache, visual turbidity, fatigue, anxiety, exercise intolerance, myalgia and chest pain may also occur.

Clinical history and physical examination must be performed to identify the potential causes for tachycardia, such as: hyperthyroidism; medicines; use of hidden substances; psychological triggers; panic attacks, and to rule out POTS, considering that both conditions share the same symptoms (Table 7).

**Table 7:** Causes that must be ruled out before diagnosing inappropriate sinus tachycardia

Medical Conditions		Physiological Conditions	Drugs/Substances
Hyperthyroidism		Physical exercise	Caffeine
Cushing disease		Emotional stress	Alcohol
Pheochromocytoma		Pain	Tobacco
Anemia		Fever	Catecholamines
Infections		Pregnancy	Vasodilators
Dehydration		Volume depletion	Substances with atropine
Cardiomyopathy			Theophylline
Panic attack			Illicit drugs
Pericarditis			Decongestants
	Mitral or aortic regurgitation		Sympathomimetics
Myocardial infarction			Thyroid-stimulating hormones
Orthostatic hypotension			

Source: author and adapted.^10^^,^^110^

Patients should be investigated for hypovolemia, which is observed in some cases. However, it is necessary to rule out structural heart disease for the diagnosis of IST. In the natural history of patients with IST, in general, there is no worsening of ventricular function due to tachycardia.^109^ However, there are rare descriptions of isolated cases of tachycardiomyopathy, challenging the assumption that IST is always a benign condition.^111^^,^^113^^,^^114^

Stress testing can be useful in documenting exaggerated tachycardia in response to exercise. Cardiovascular autonomic tests, including HR response to Valsalva maneuver, deep breathing and orthostatic position, as well as HR variability and baroreflex sensitivity, have not shown clinical usefulness and, therefore, should not be routinely employed.^11^

**Inappropriate Sinus Tachycardia (IST)**

It is characterized when the resting heart rate is greater than 100 bpm and the average HR is greater than 90 bpm on 24-h Holter in adolescents and young adults. It occurs more commonly in women, without a reasonable cause. It is associated with various severe and often debilitating symptoms, especially palpitations, dizziness and syncope.

People with IST usually experience a significant loss of quality of life. There are no placebo-controlled prospective clinical studies for the therapeutic interventions used in the treatment, and some symptoms may persist despite HR control.

There is some evidence that ivabradine, at a dose of 5 to 7.5 mg, twice a day, can improve quality of life. ^115^^,^^116^^,^^117^ In addition, it appears that ivabradine may have benefits when associated with beta-blockers (metoprolol).^118^

Beta-blockers alone are not useful and can cause side effects. Other treatments have been proposed, such as: drugs such as fludrocortisone; clonidine; erythropoietin; non-pharmacological measures, such as elastic compression stockings; physical exercises and, rarely, radiofrequency ablation, which may pose risks of sinus node injury, requiring the implantation of a cardiac pacemaker.^119^ Patients with IST usually require special attention and lifestyle changes.

## Note

Part II of this article, which describes clinical and cardiovascular symptoms, methods of investigation and treatment, will continue in the next issues of the journal.
